# Supplementary material for: Investigating the origin of subtelomeric and centromeric AT-rich elements in Aspergillus flavus
Source: PLoS One. 2023 Feb 9;18(2):e0279148. doi: 10.1371/journal.pone.0279148 (PMC9910759; doi:10.1371/journal.pone.0279148)
Supplement: S2 Fig — Strains were compared as described in S1 Fig. The tree is rooted in CA14 AT insertion 1–4 (S5 Table). The scale for 0.01% homology difference is shown on the bottom right. (PDF) [file pone.0279148.s002.pdf]

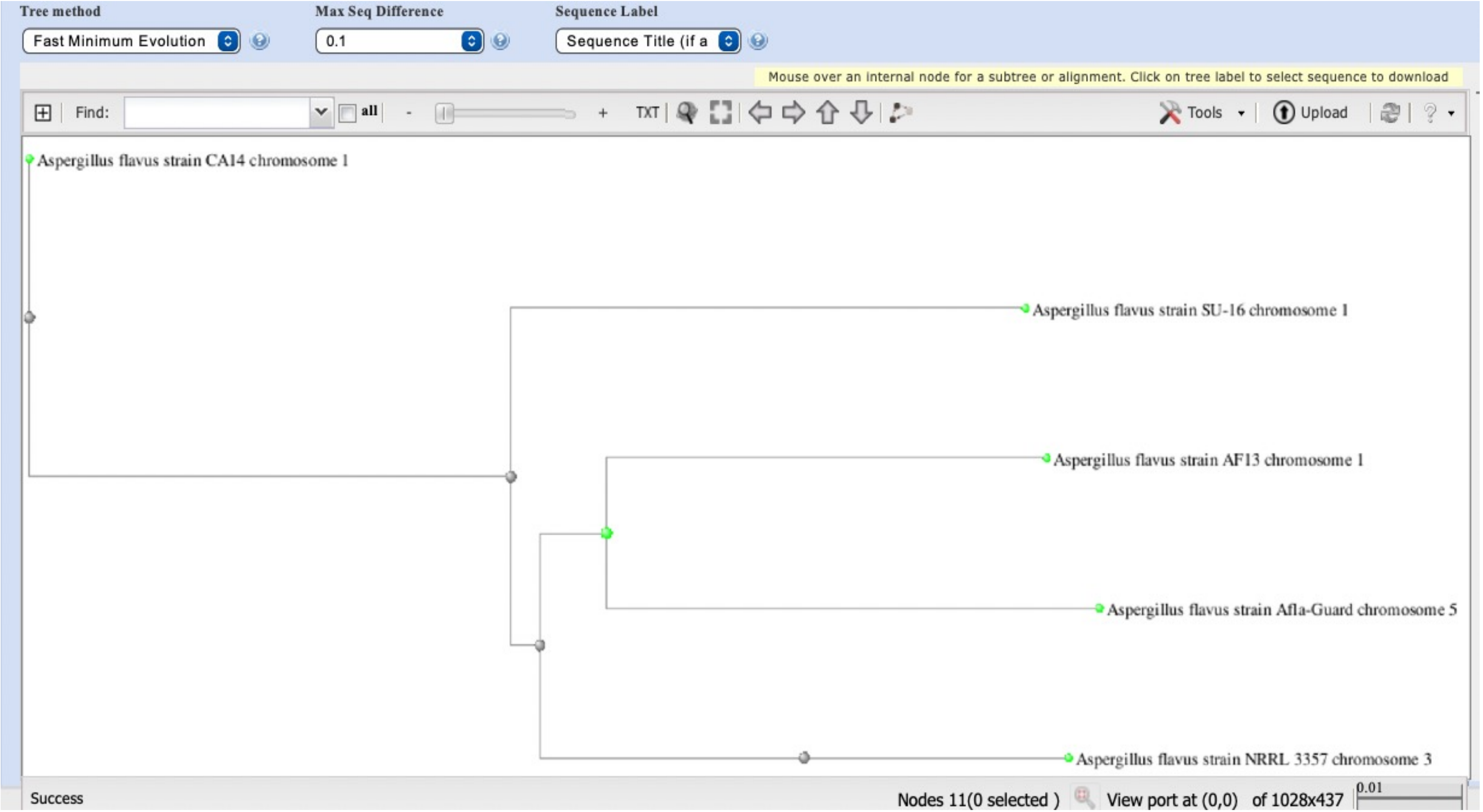

Supplementary Figure 2: CA14 Chr1-4-AT Indel Phylogenetic Comparisons with Other Strain Displaying Strains at the 90% Homology Level. The scale for 0.01% homology difference is shown on the bottom right. Tree is rooted in CA14.
