# Supplementary material for: Investigating the origin of subtelomeric and centromeric AT-rich elements in Aspergillus flavus
Source: PLoS One. 2023 Feb 9;18(2):e0279148. doi: 10.1371/journal.pone.0279148 (PMC9910759; doi:10.1371/journal.pone.0279148)
Supplement: S3 Fig — A) Class A repeat organization measured by homology to NRRL ATEs 1–1 and 3–3; B) Class B repeat organization measured by homology to NRRL ATE 1–2; C) Class C repeat organization as assayed by homology to 3357 ATE 1–4; D); Class D repeat organization as assayed by homology to 3357 ATE 1–6; E) Class E repeat organization as assayed by homology to 3357 ATEs 1–6; F) Class F repeat organization as assayed by homology to 3357 ATE 1–3; G) Class G repeat organization as assayed by homology to CA14 ATE 3–3; H) Class H repeat organization as assayed by homology to 3357 ATE 4–1 and 6–1; I) Class I repeat organization as assayed by homology to 3357 ATE 3–1 and 7–1; J) Class J repeat organization as assayed by homology to 3357 ATE 8–2; K) Class K repeat organization as assayed by homology to SU-16 ATE 5–4. L) Class L repeat organization as assayed by homology to SU-16 ATE 8–1. Each class corresponds to the classes given by the superscripts in Table 2A, 2B, and 2C. In 3B-3E, green line, homology to the relevant ATE; the orange line, Gypsy elements; dark orange line, LTR 1 and LTR 2; red line, Mariner elements; dark red line, Mariner 2N1 and Mariner 5; blue line, TAD 1 elements. The AO designation refers those TEs identified in Censor as A. oryzae derivatives. The AF designation is used for those Gypsy elements identified in A. flavus by BLAST querying for homologs to the Gypsy1 AO, Gypsy 2 AO, and Gypsy 4 AO elements. Some cryptic TEs were also identified by the homology search (e.g., the additional Mariner 1 elements in SU-16 ATE 5–3 and CA14 ATE 1–2). In Classes A and F-L, blue lines indicate homology to the indicated ATE. In the case of 3H, the number in parenthesis of the blue line indicates the position of homology within NRRL ATE 4–1. Relative directionality of homologous species in 3A and 3F-3L is indicated by the arrowhead. (PDF) [file pone.0279148.s003.pdf]

Supplementary Figure 3A: Class A Repeat Organization

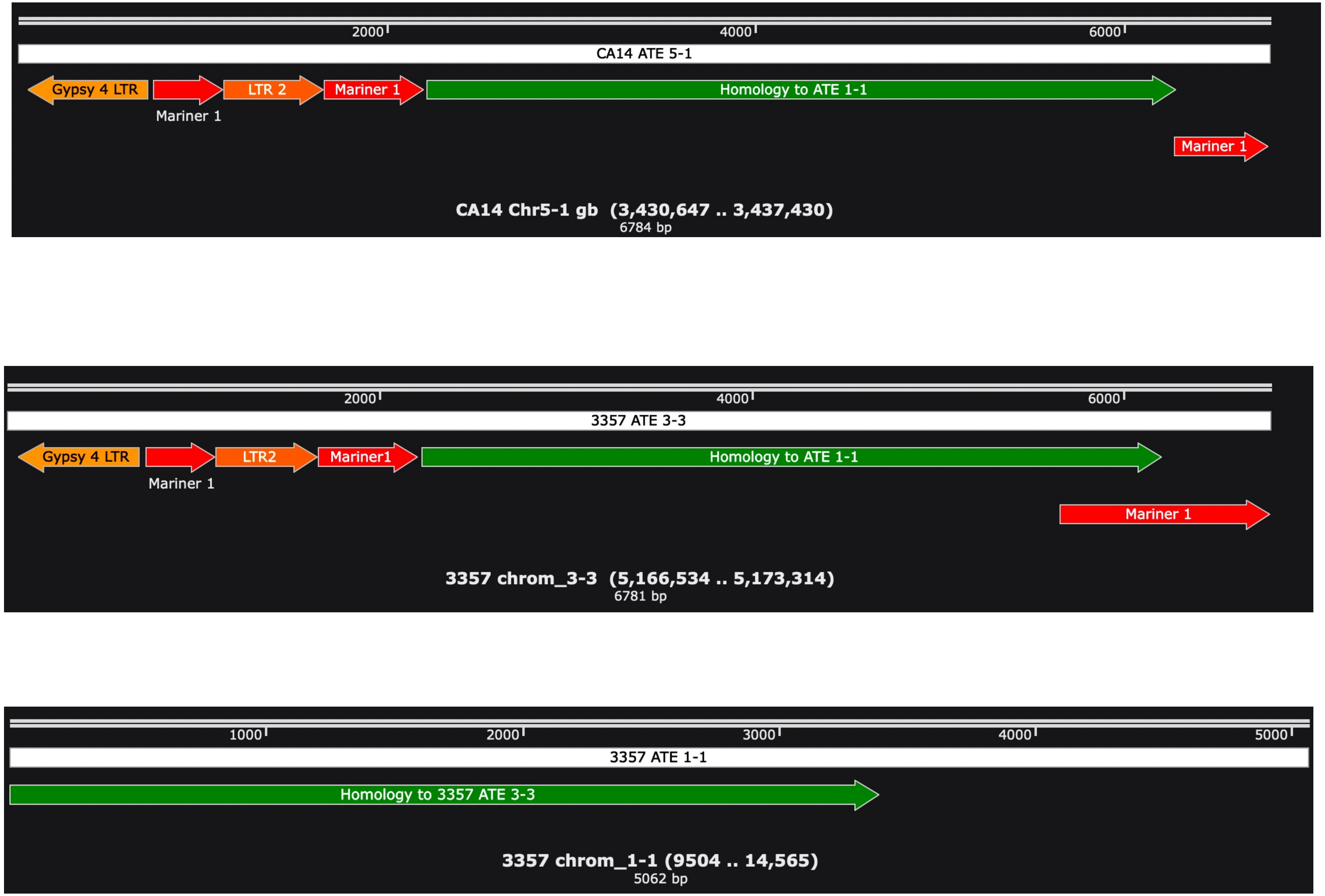

Supplementary Figure 3B: Class B Repeat Organization

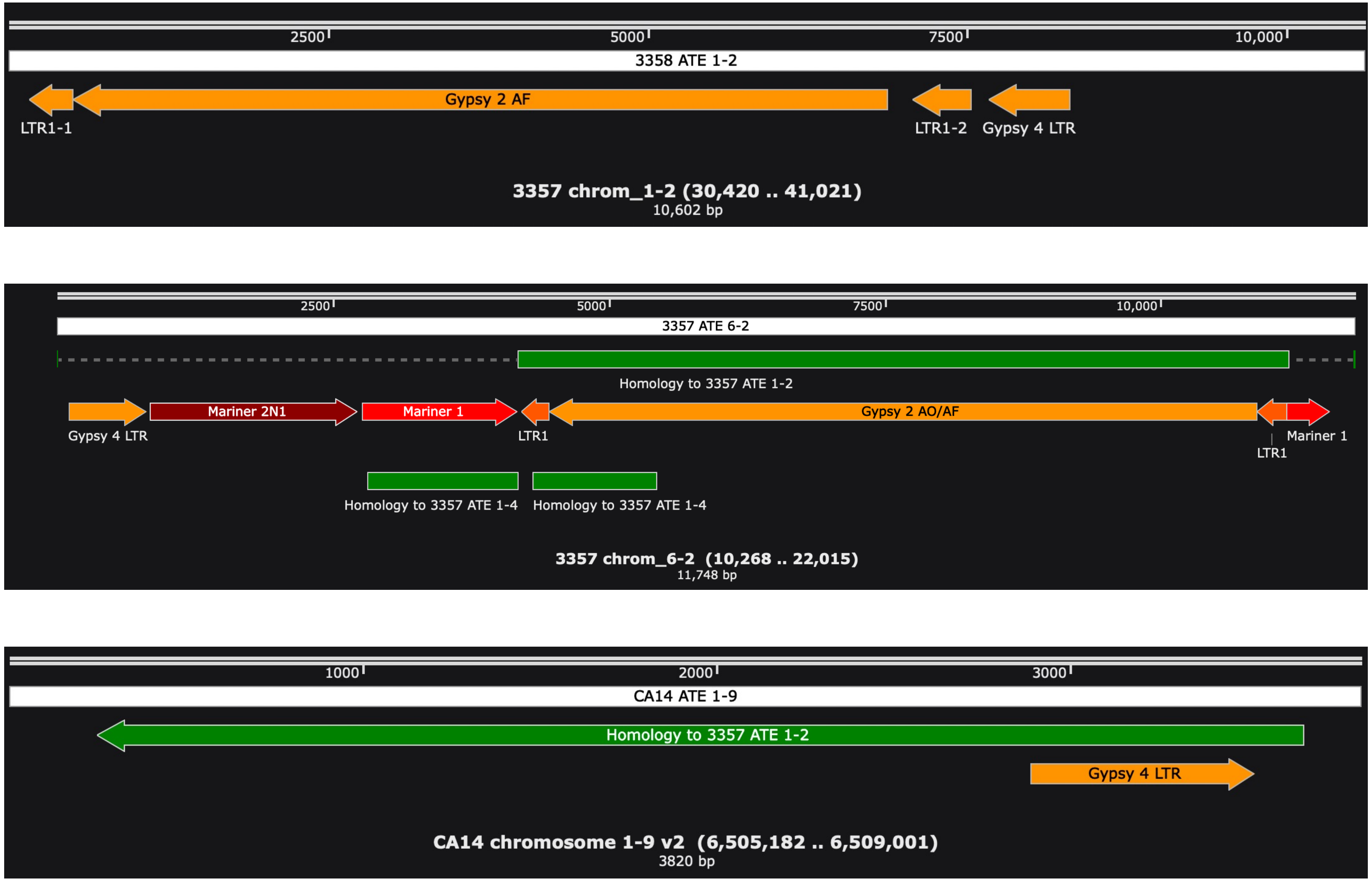

Supplementary Figure 3B, continued

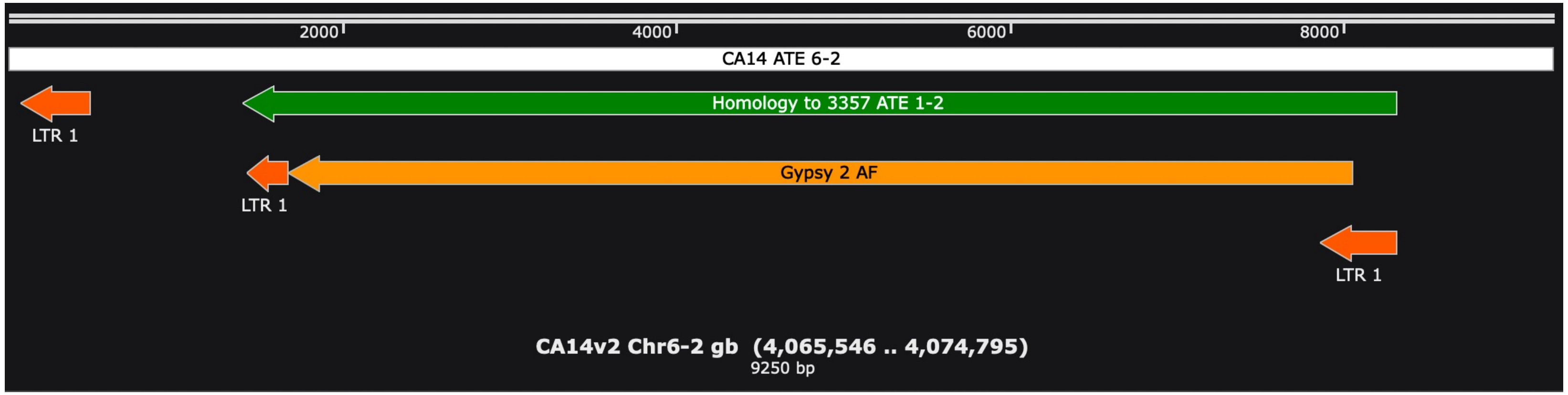

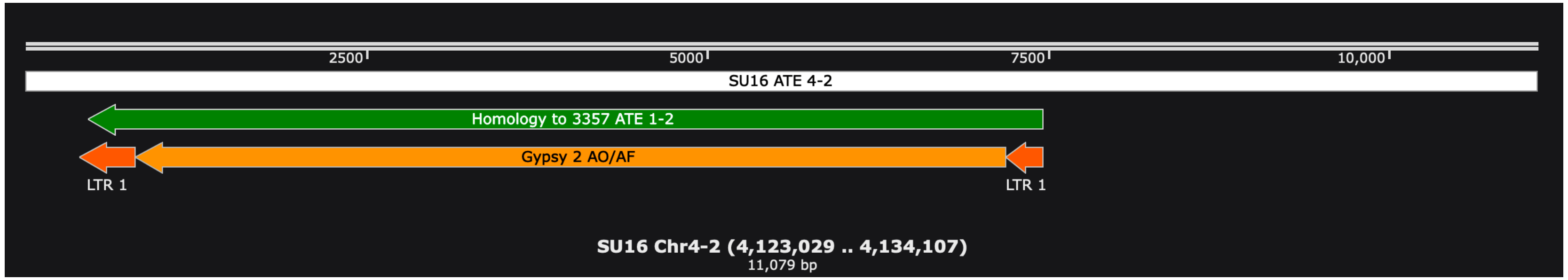

Supplementary Figure 3C: Class C Repeat Organization

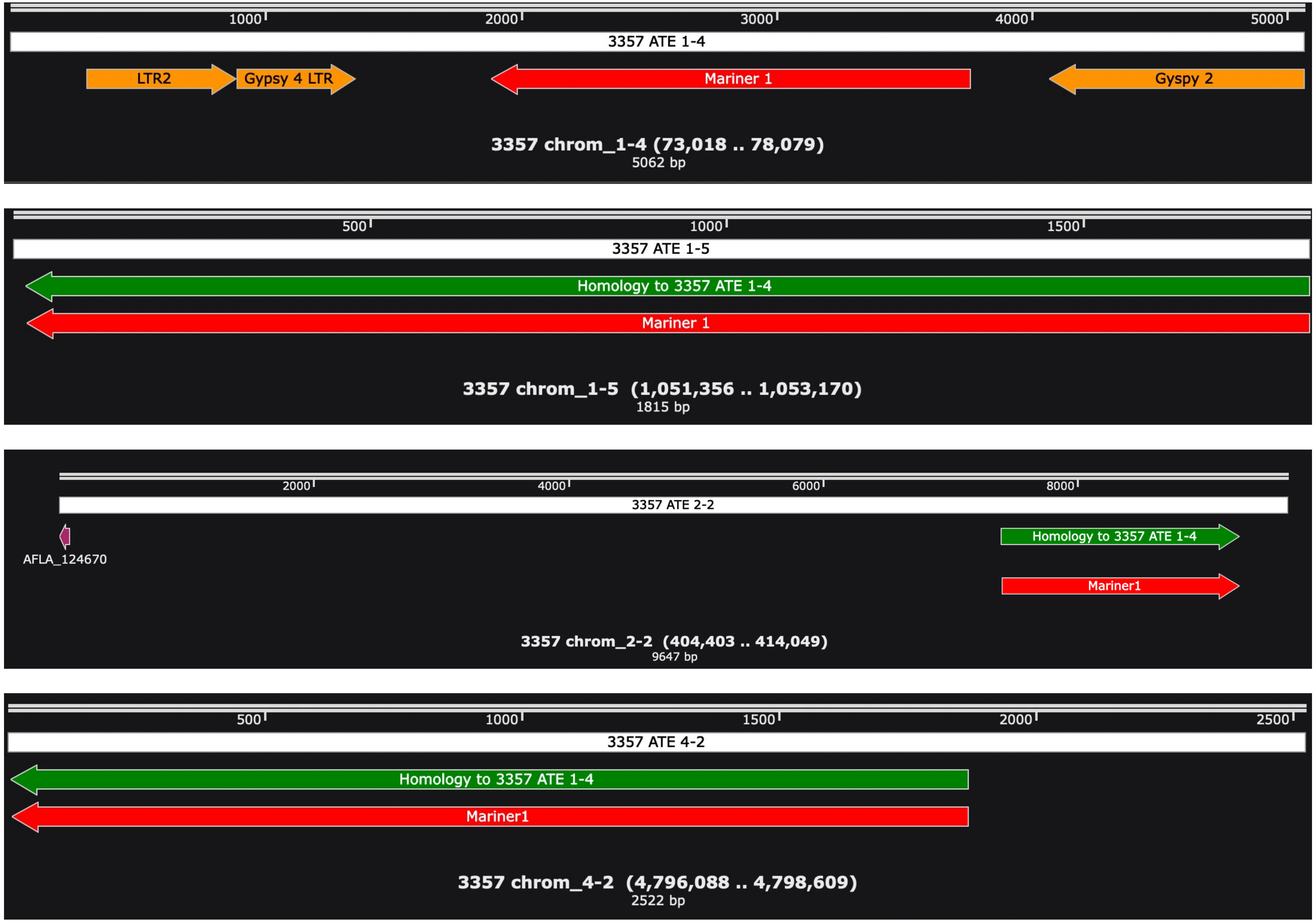

Supplementary Figure 3C, continued

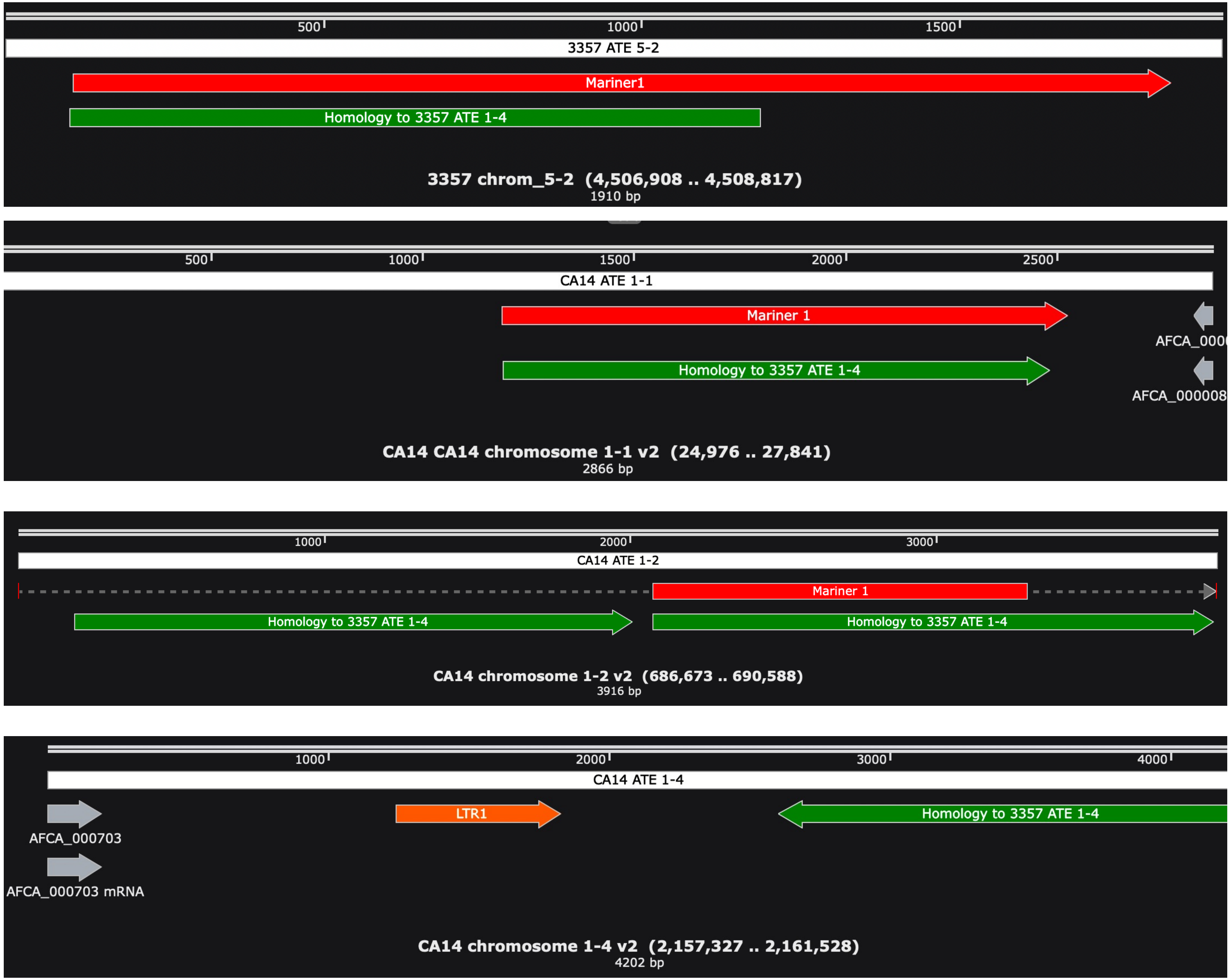

Supplementary Figure 3C, continued

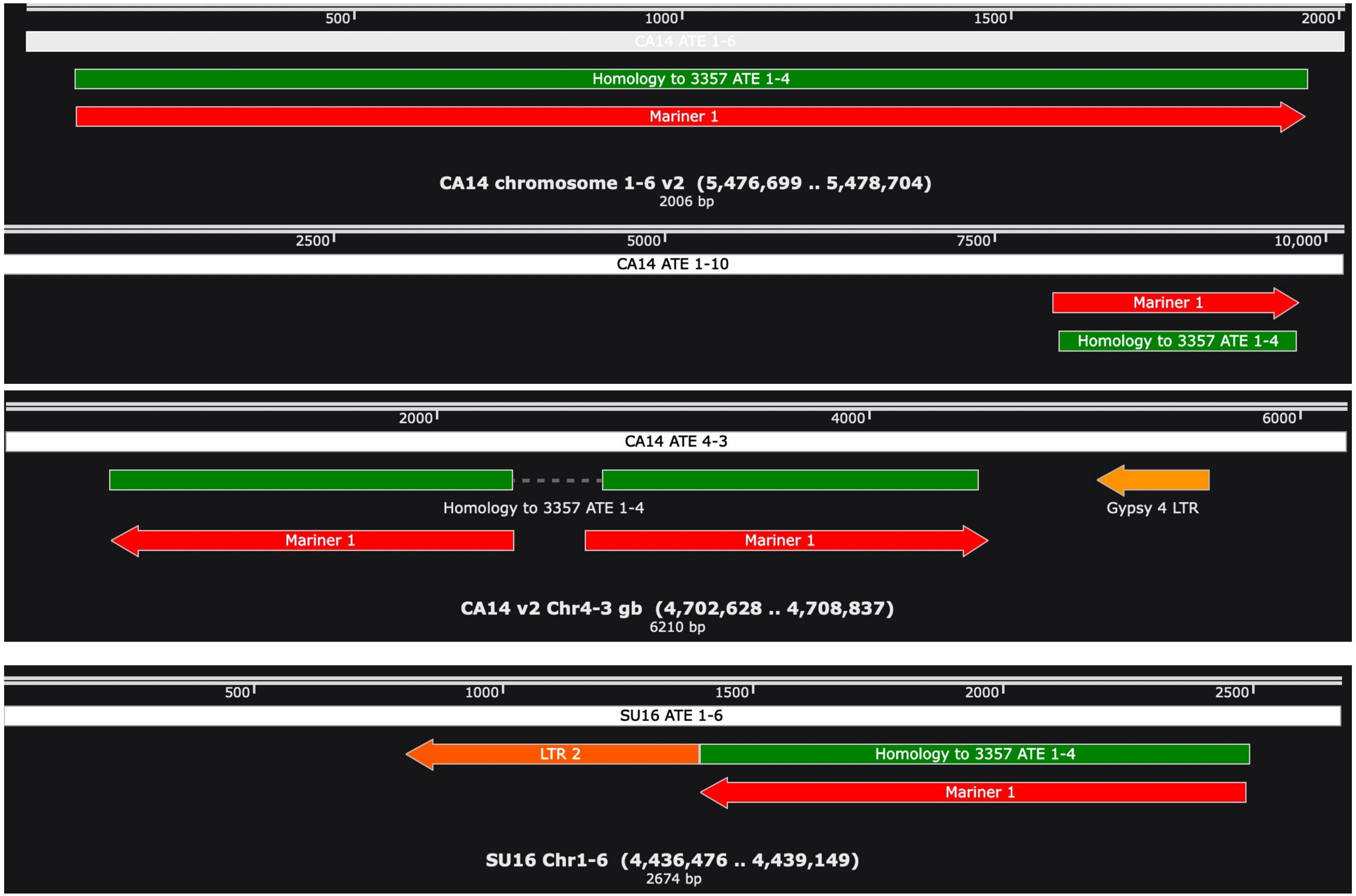

Supplementary Figure 3C, continued

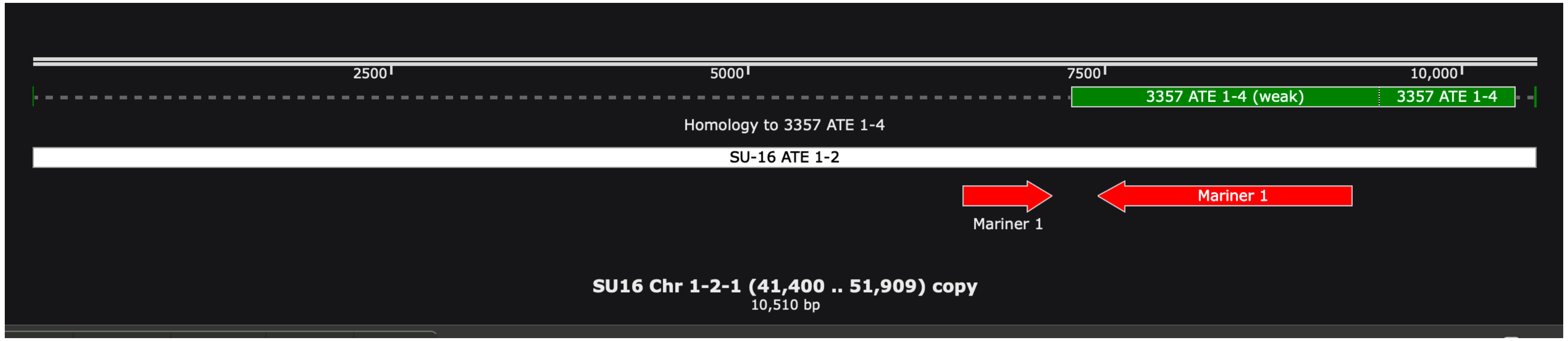

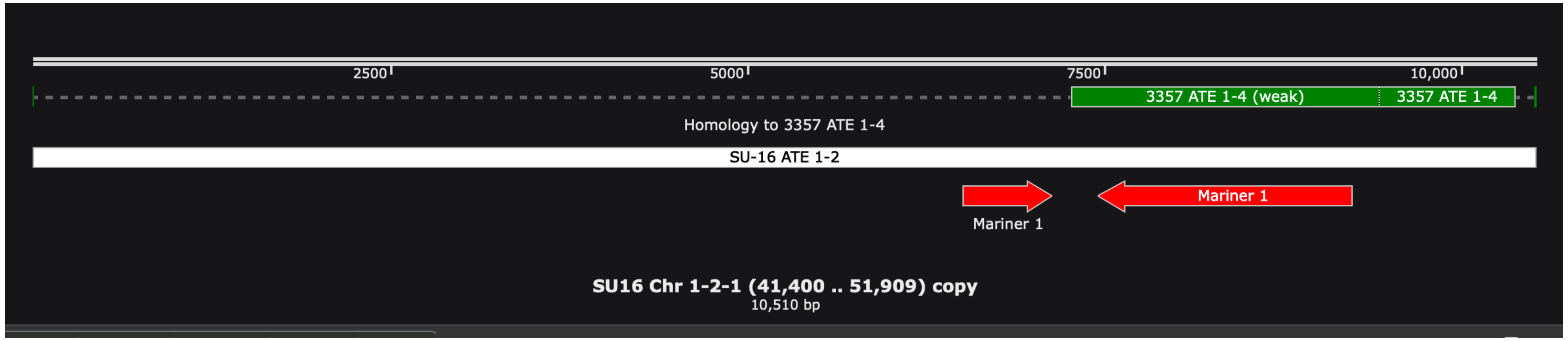

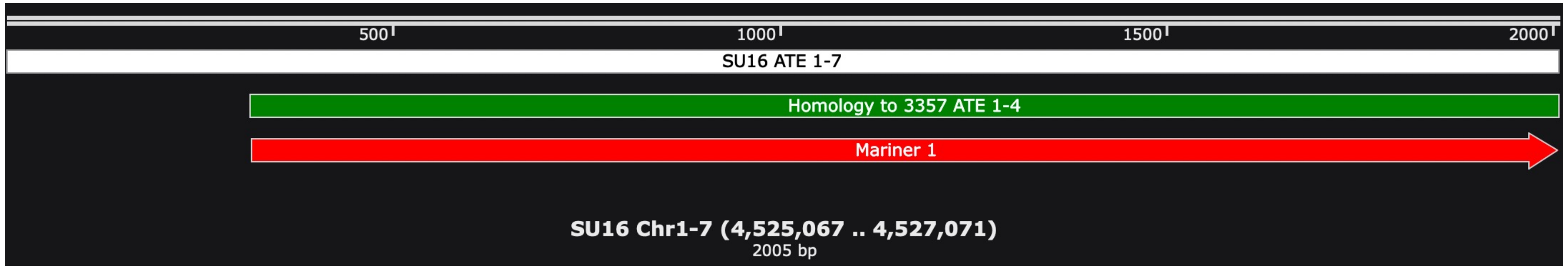

Supplementary Figure 3C, continued

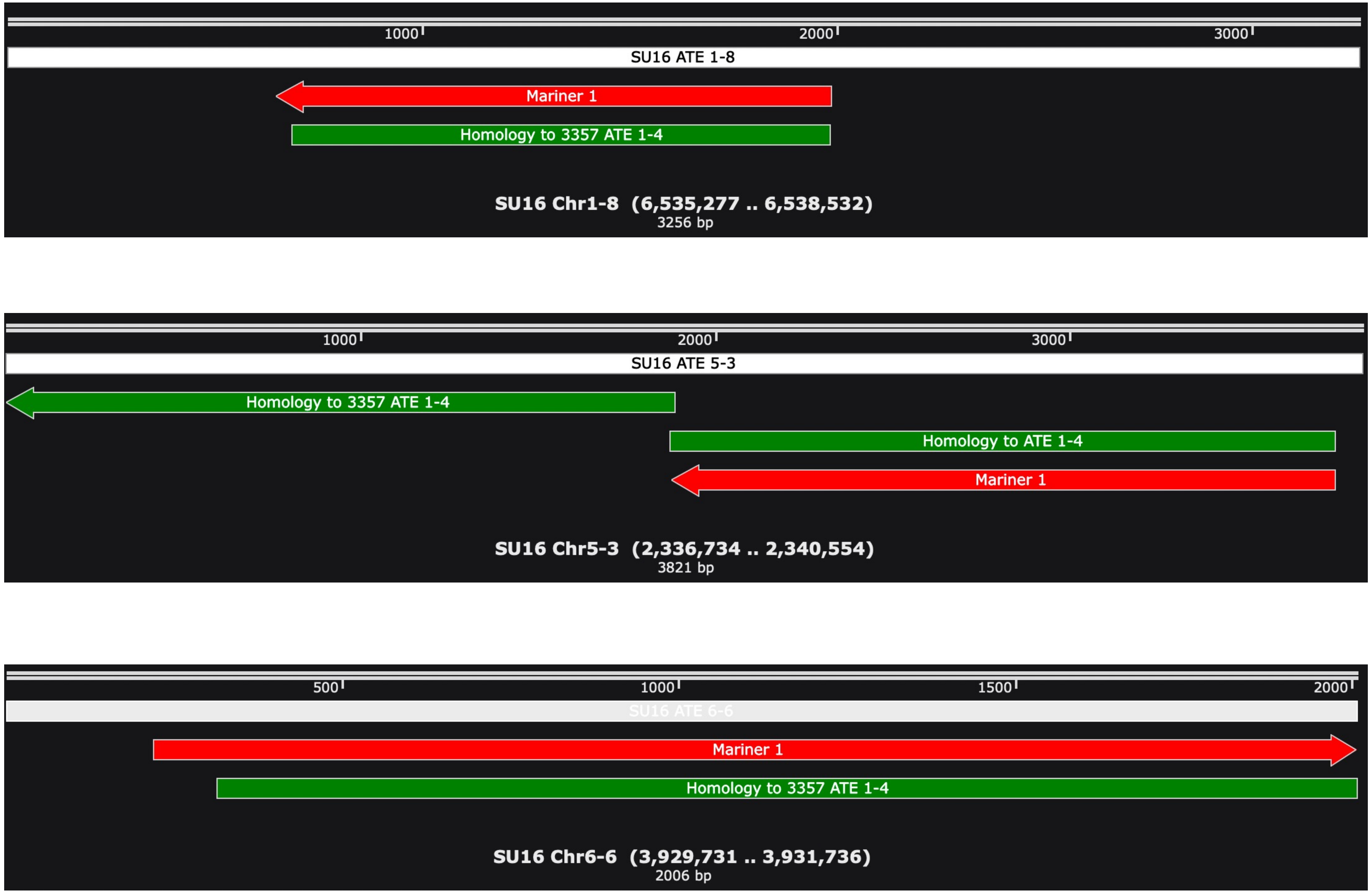

Supplementary Figure 3C, continued

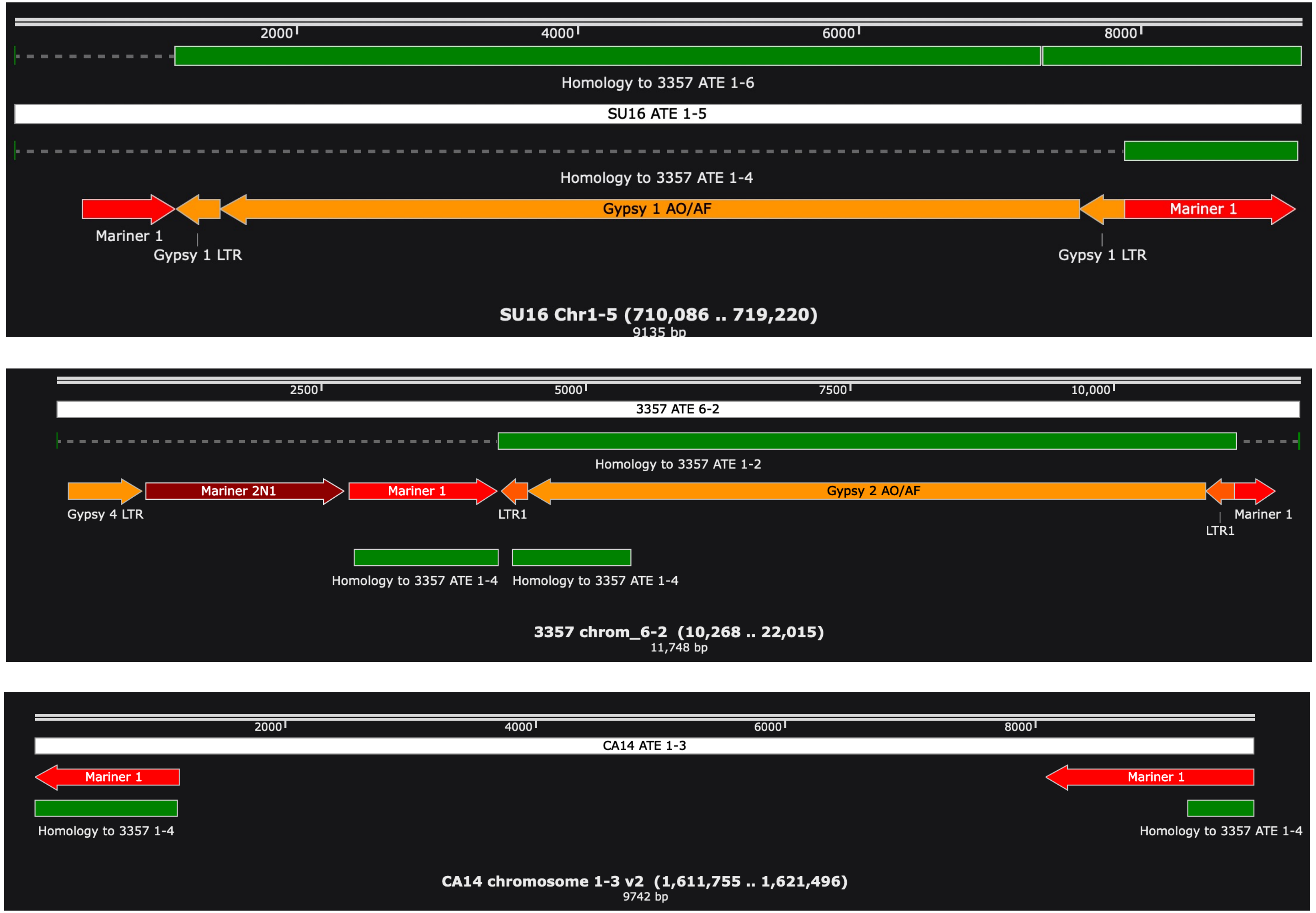

Supplementary Figure 3D: Class D Repeat Organization

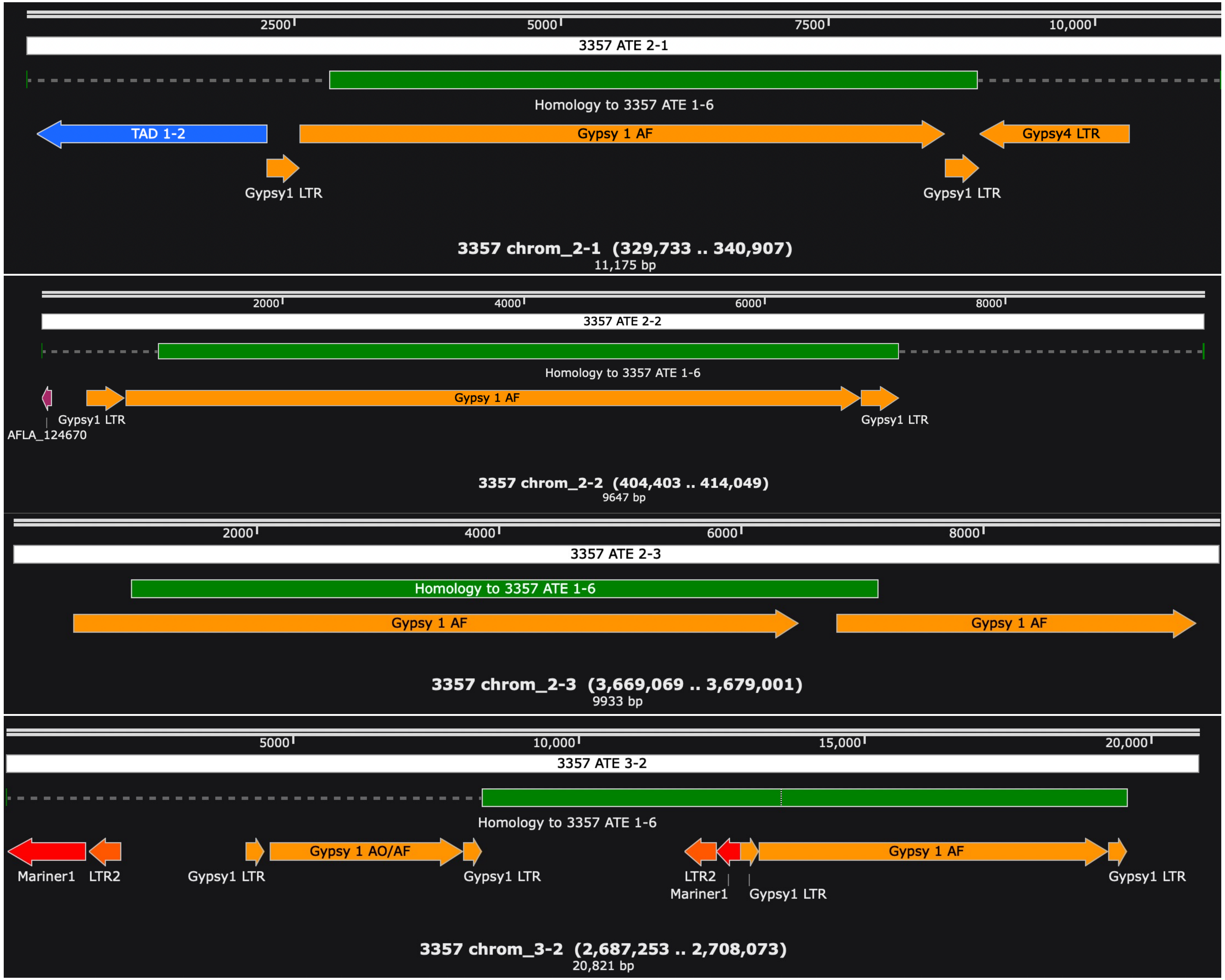

Supplementary Figure 3D, continued.

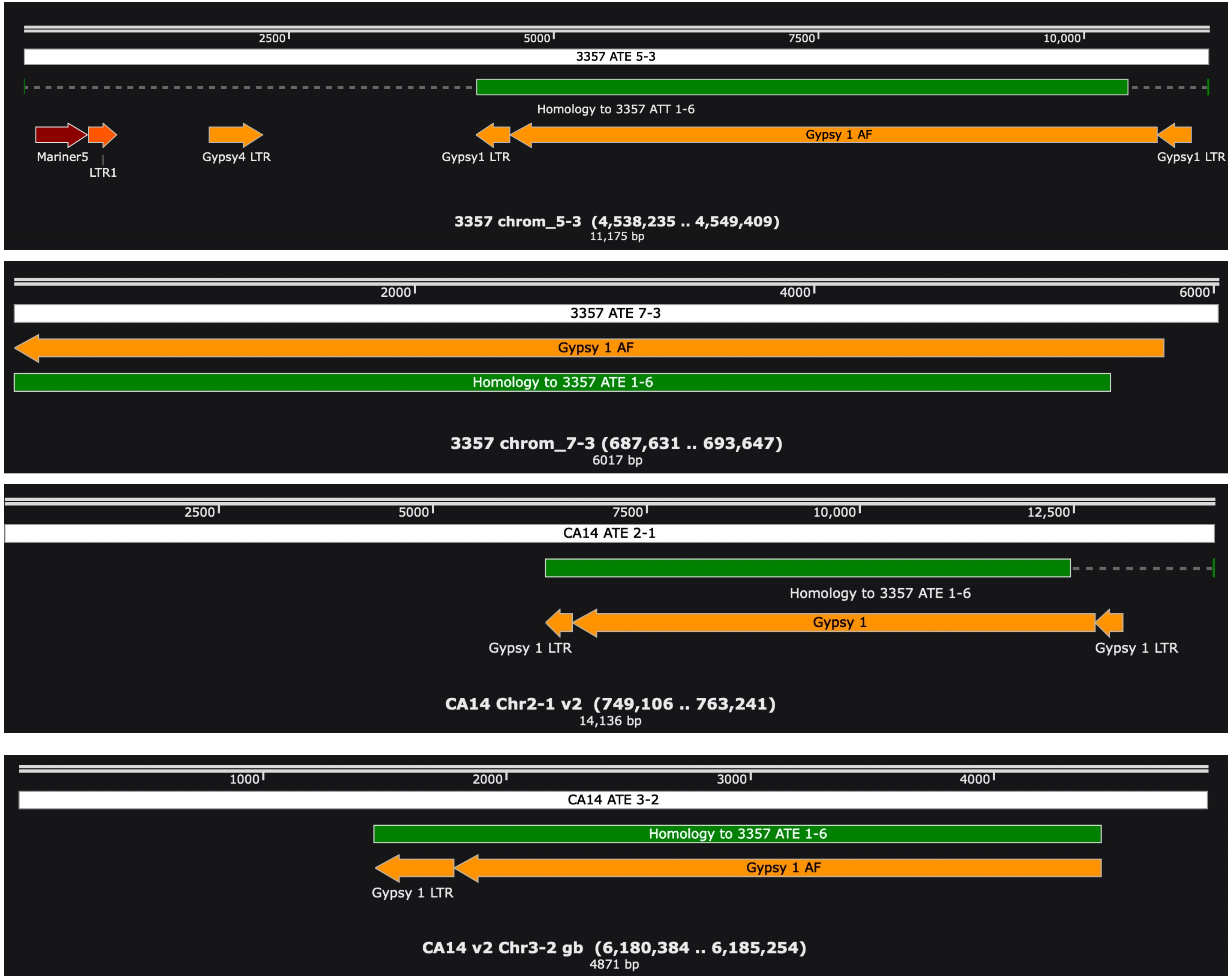

Supplementary Figure 3D, continued.

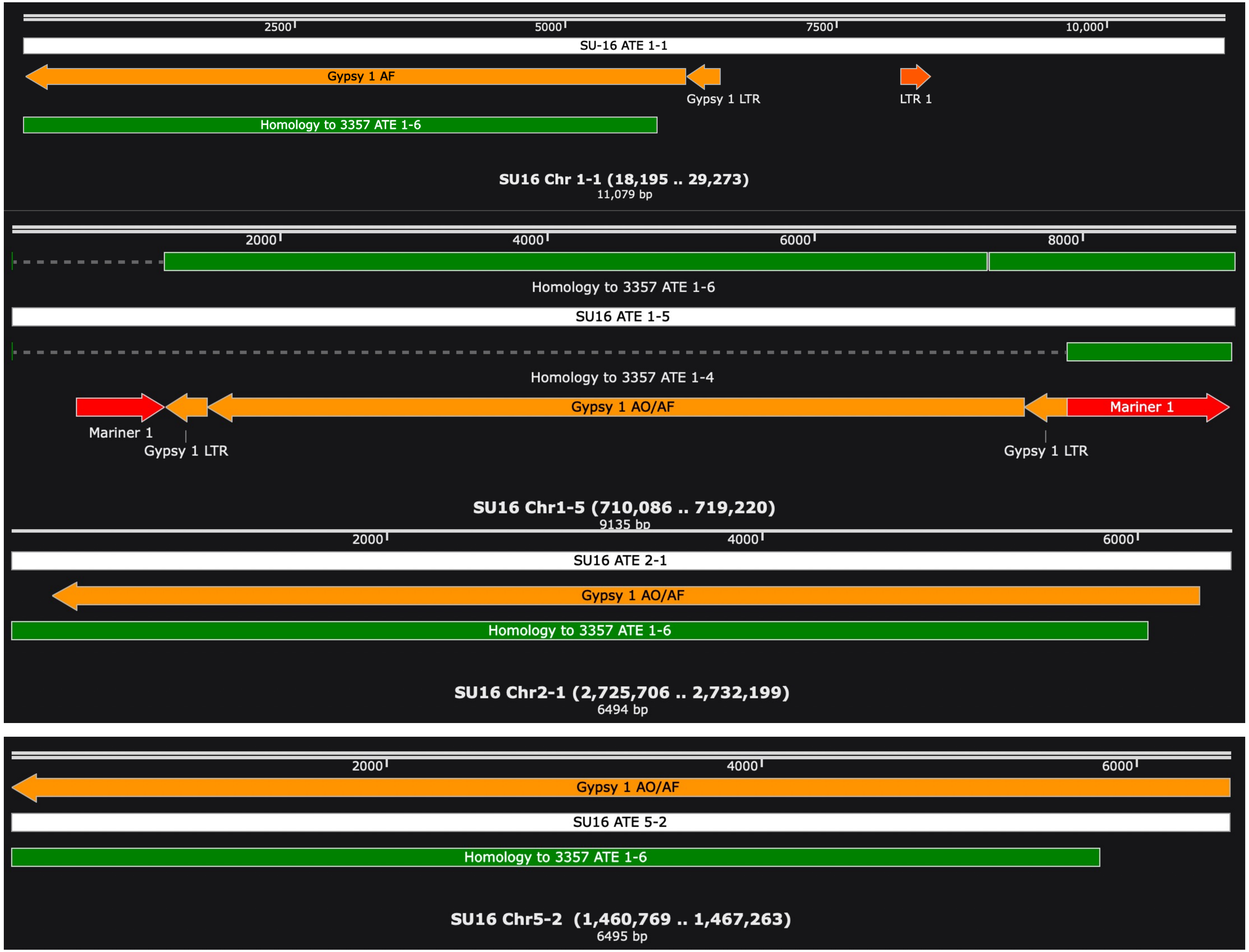

Supplementary Figure 3D, continued.

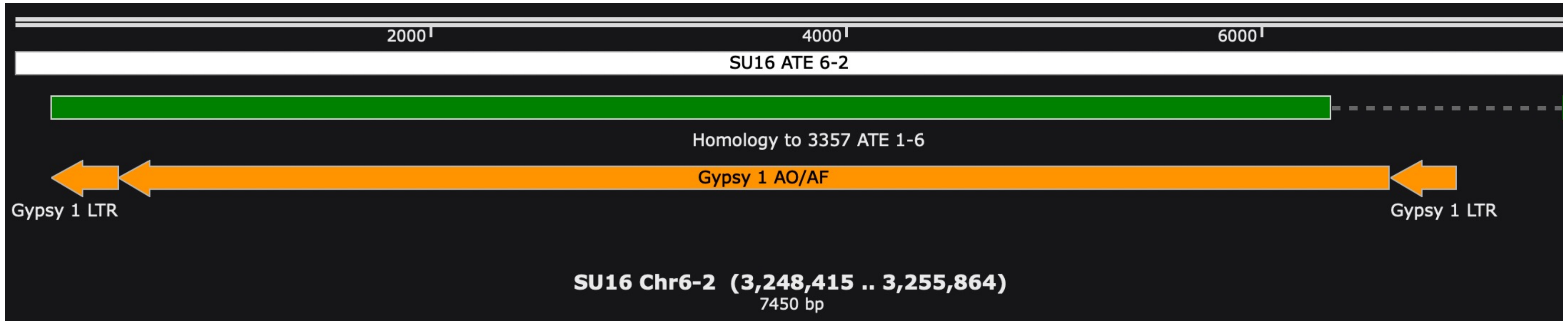

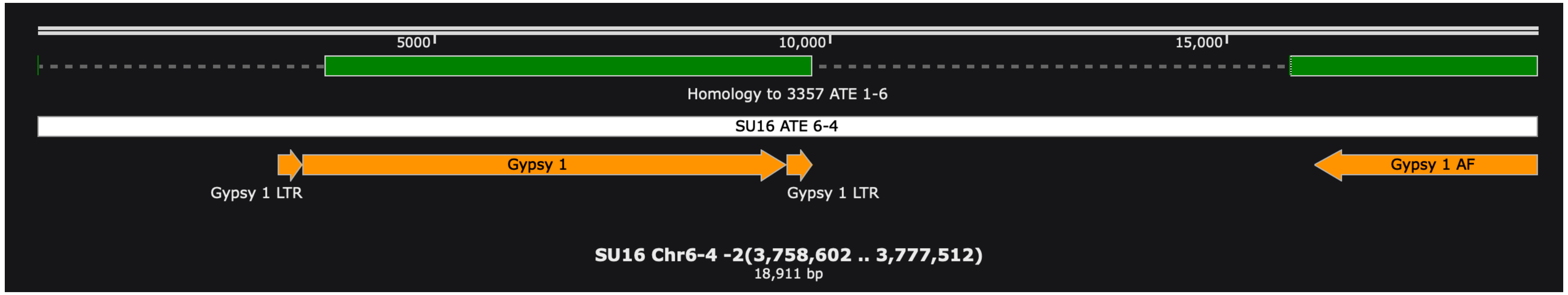

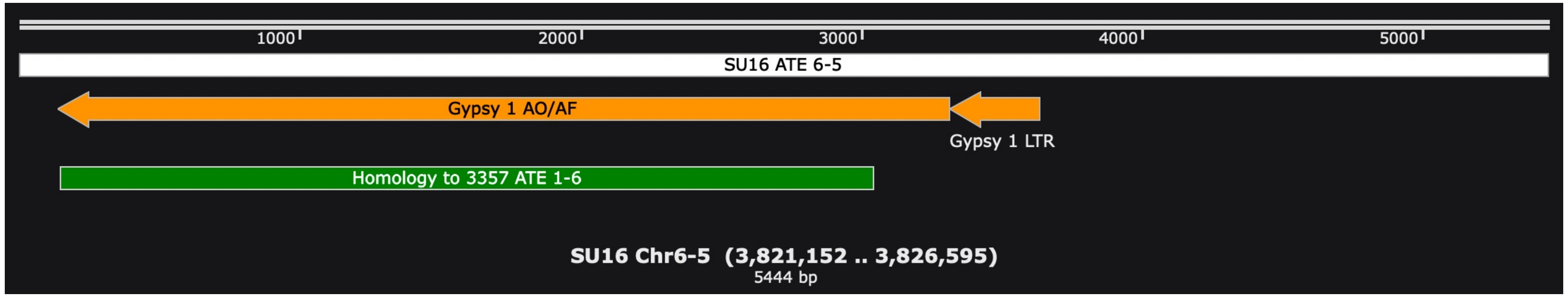

Supplementary Figure 3E: Class E Repeat Organization

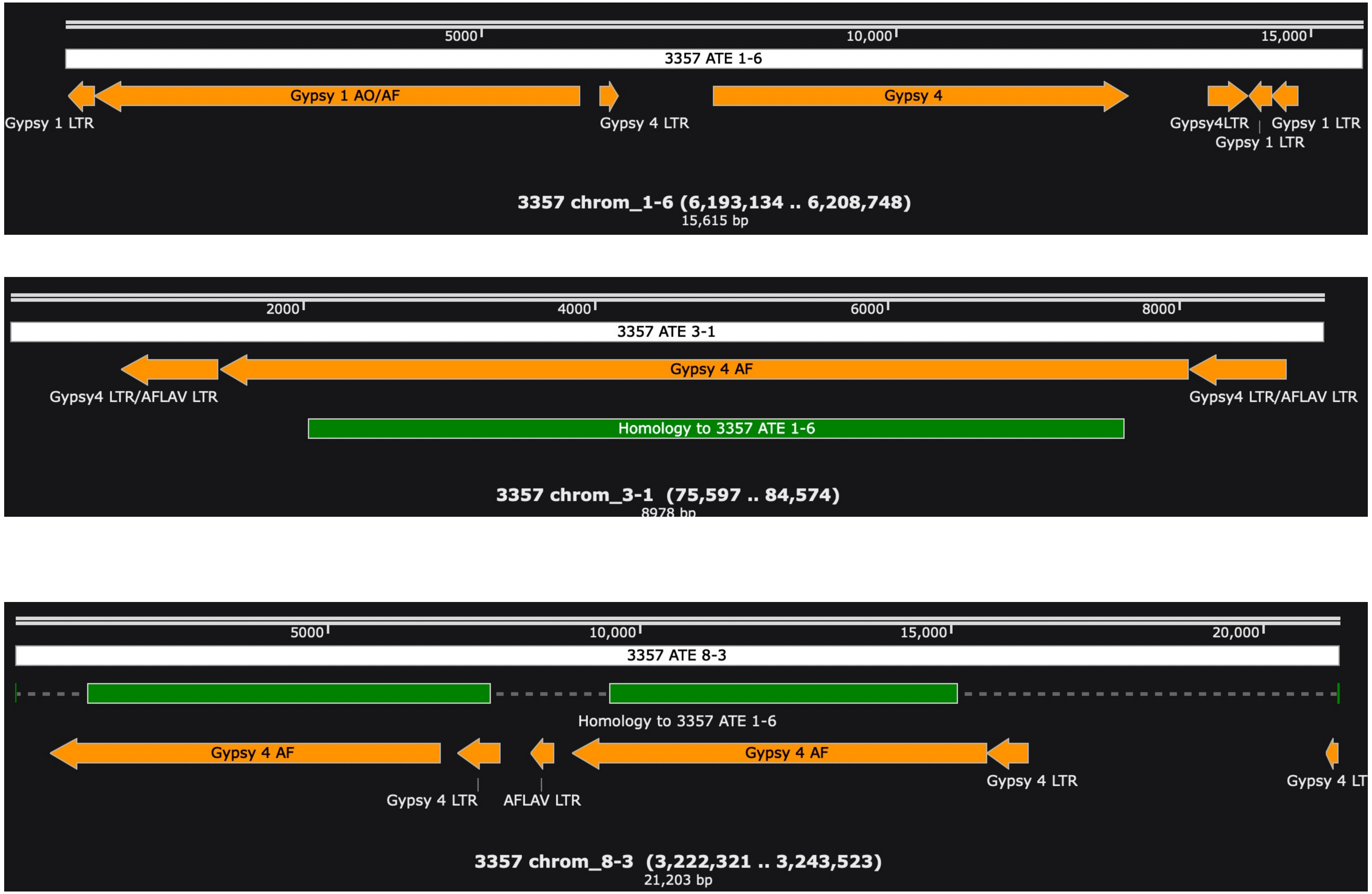

Supplementary Figure 3E, continued

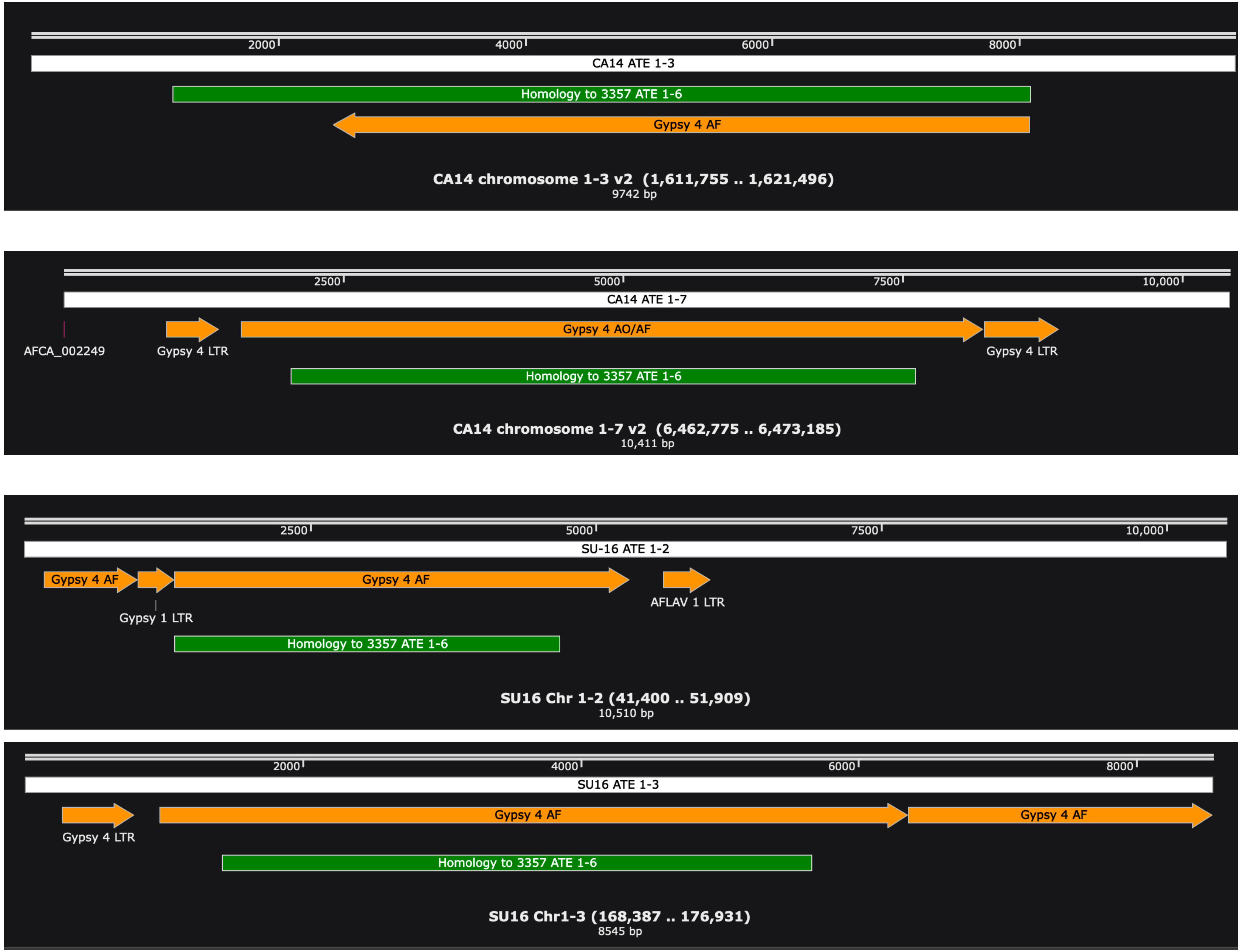

Supplementary Figure 3E, continued

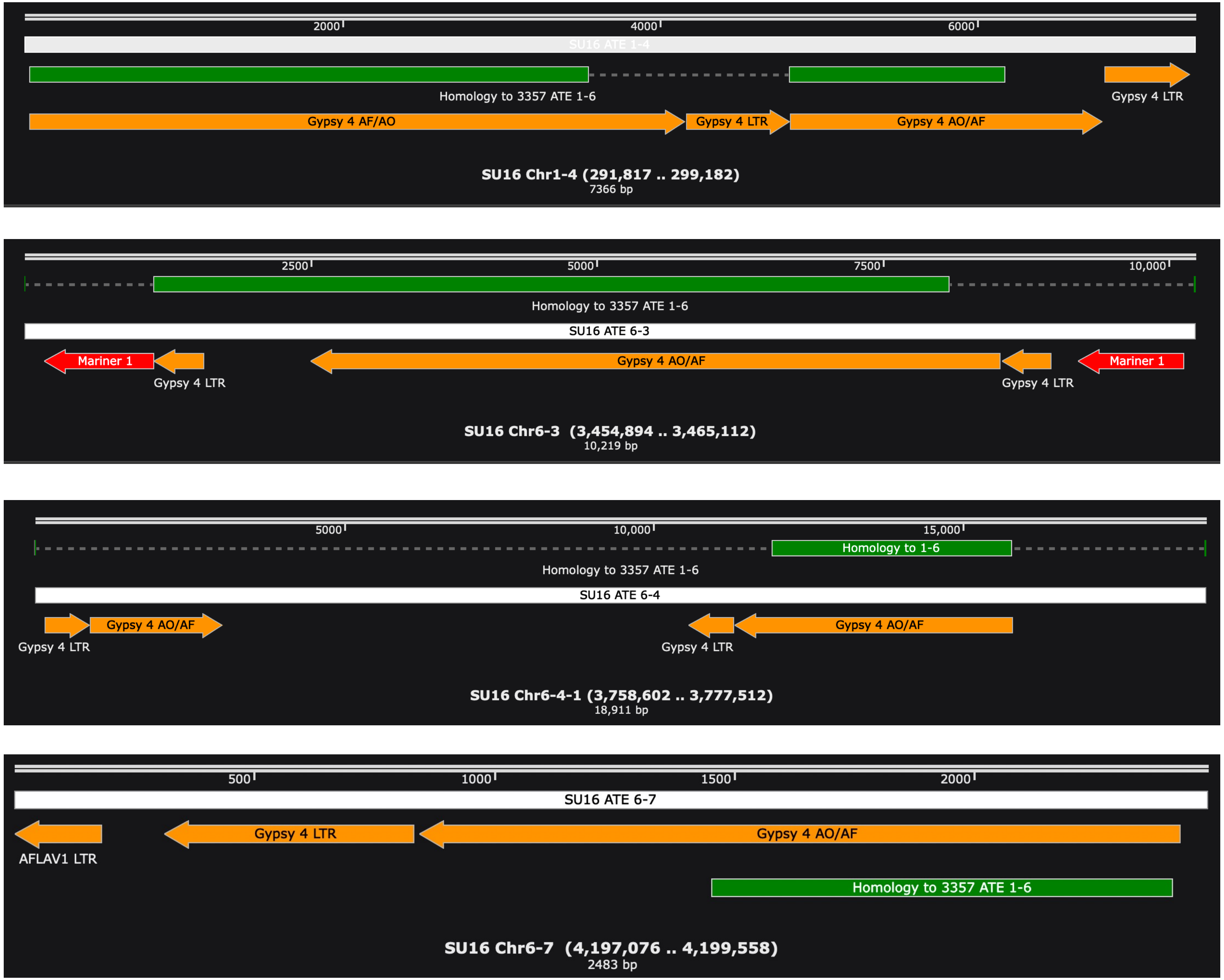

Supplementary Figure 3F: Class F Repeat Organization

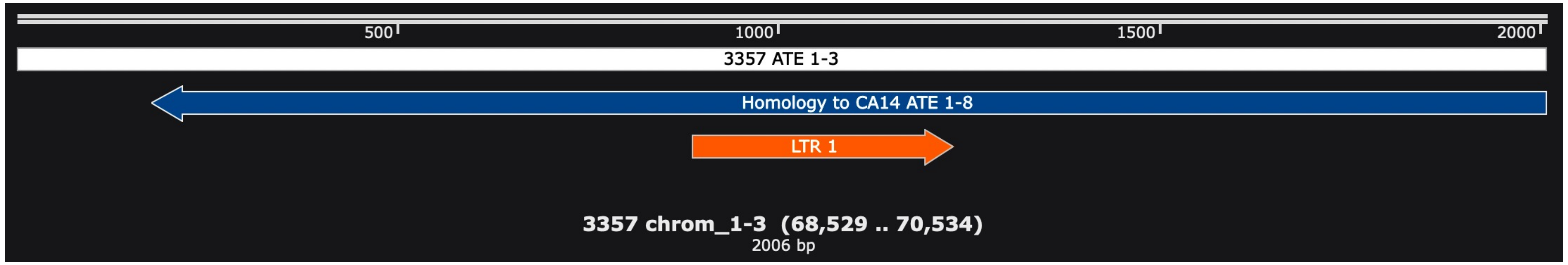

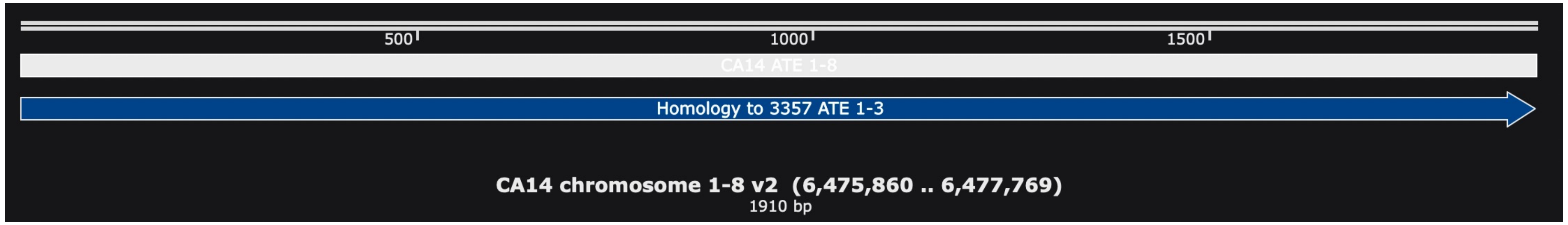

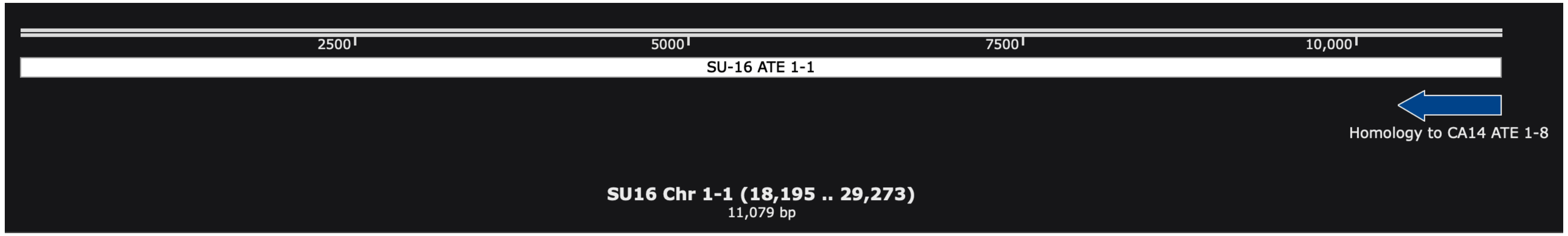

Supplementary Figure 3G: Class G Repeat Organization

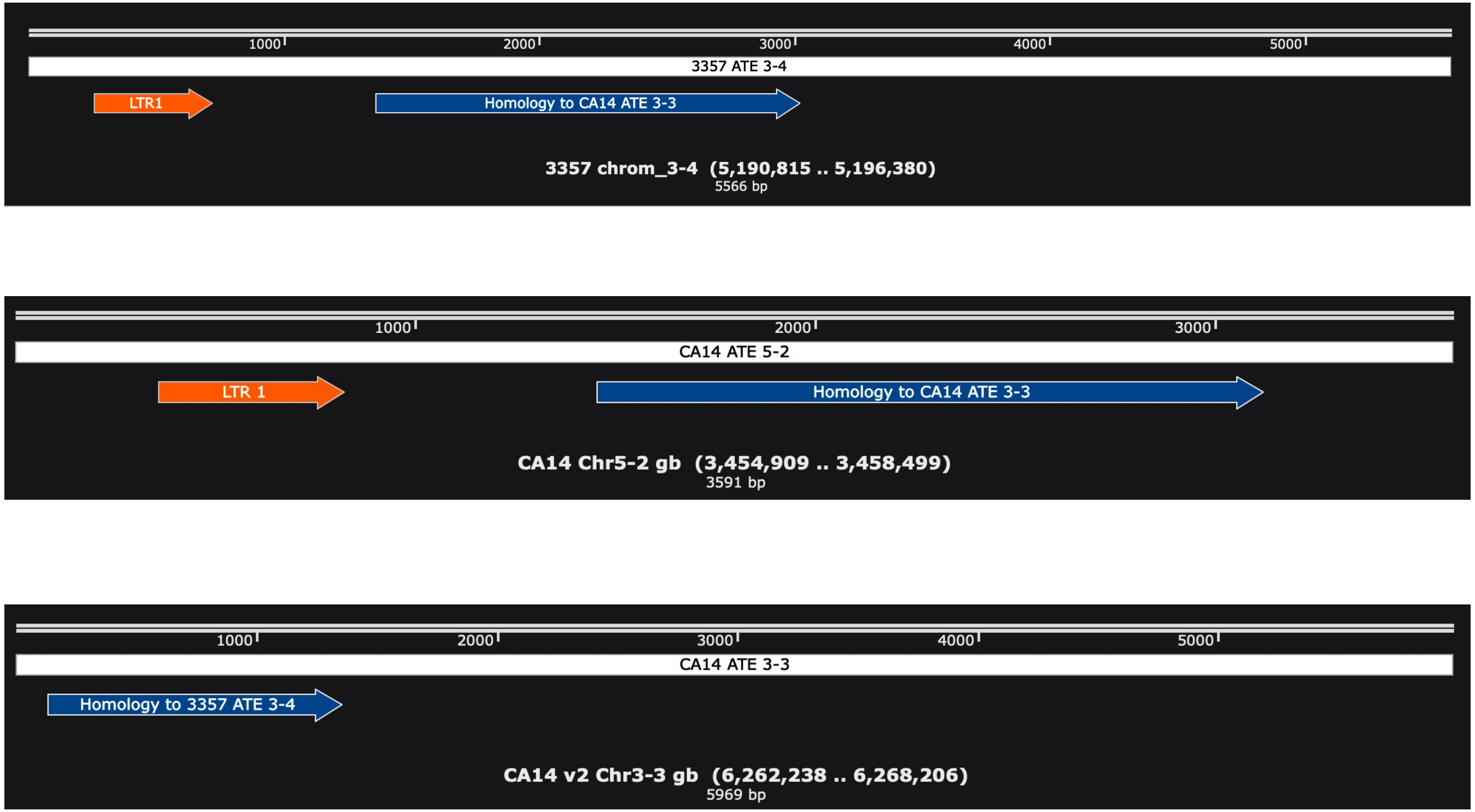

Supplementary Figure 3H: Class **H** Repeat Organization

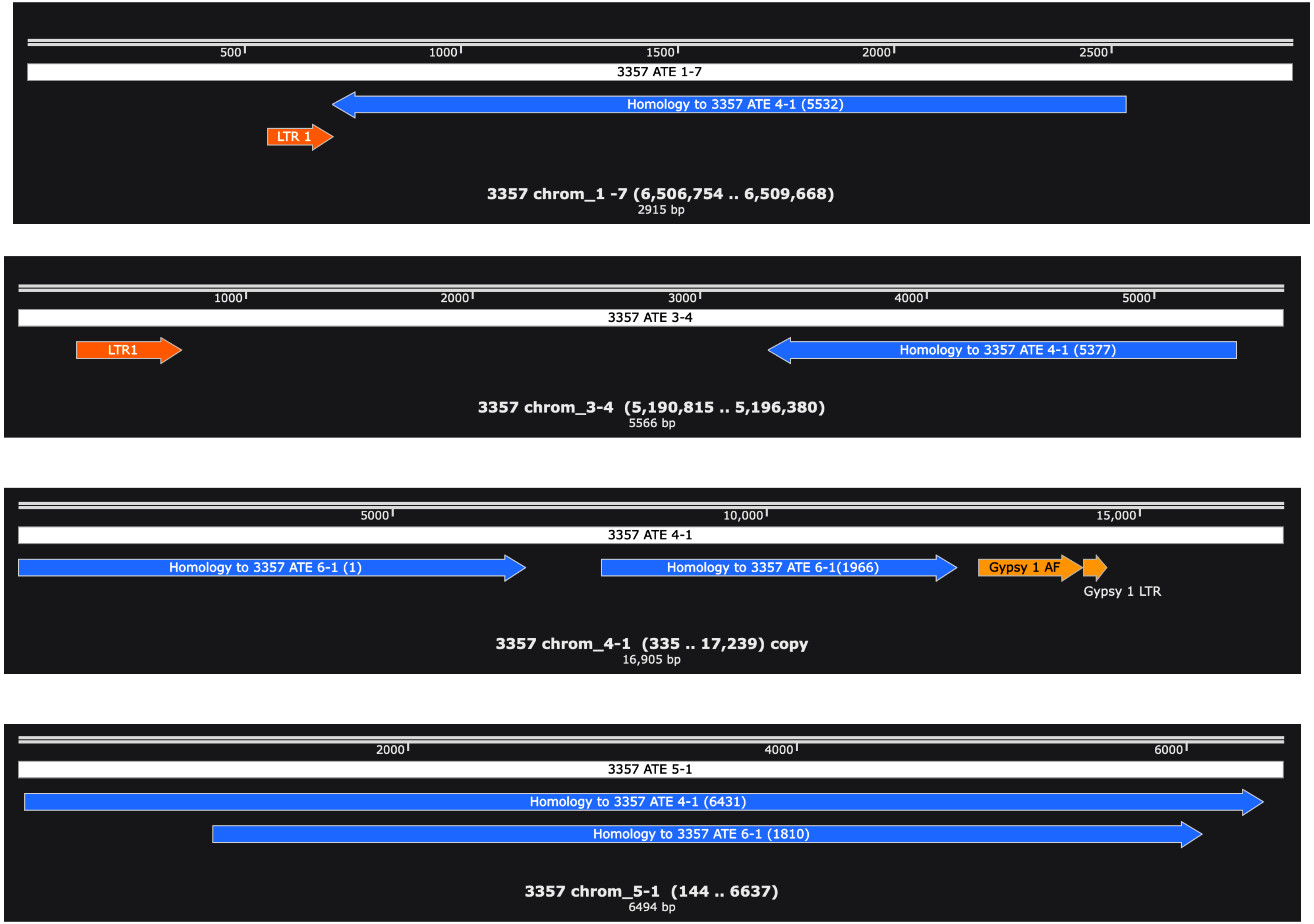

Supplementary Figure 3H, continued

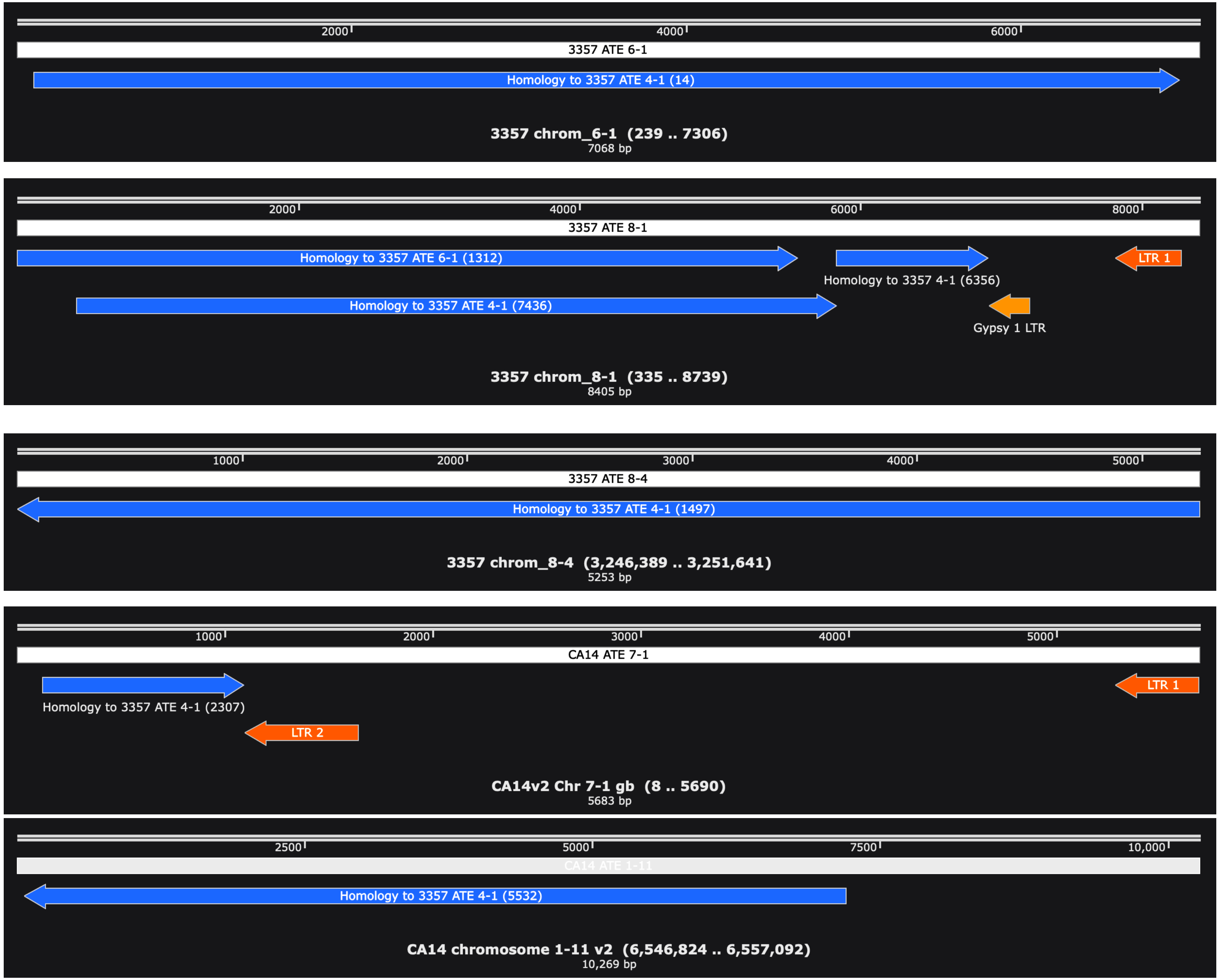

Supplementary Figure 3H, continued

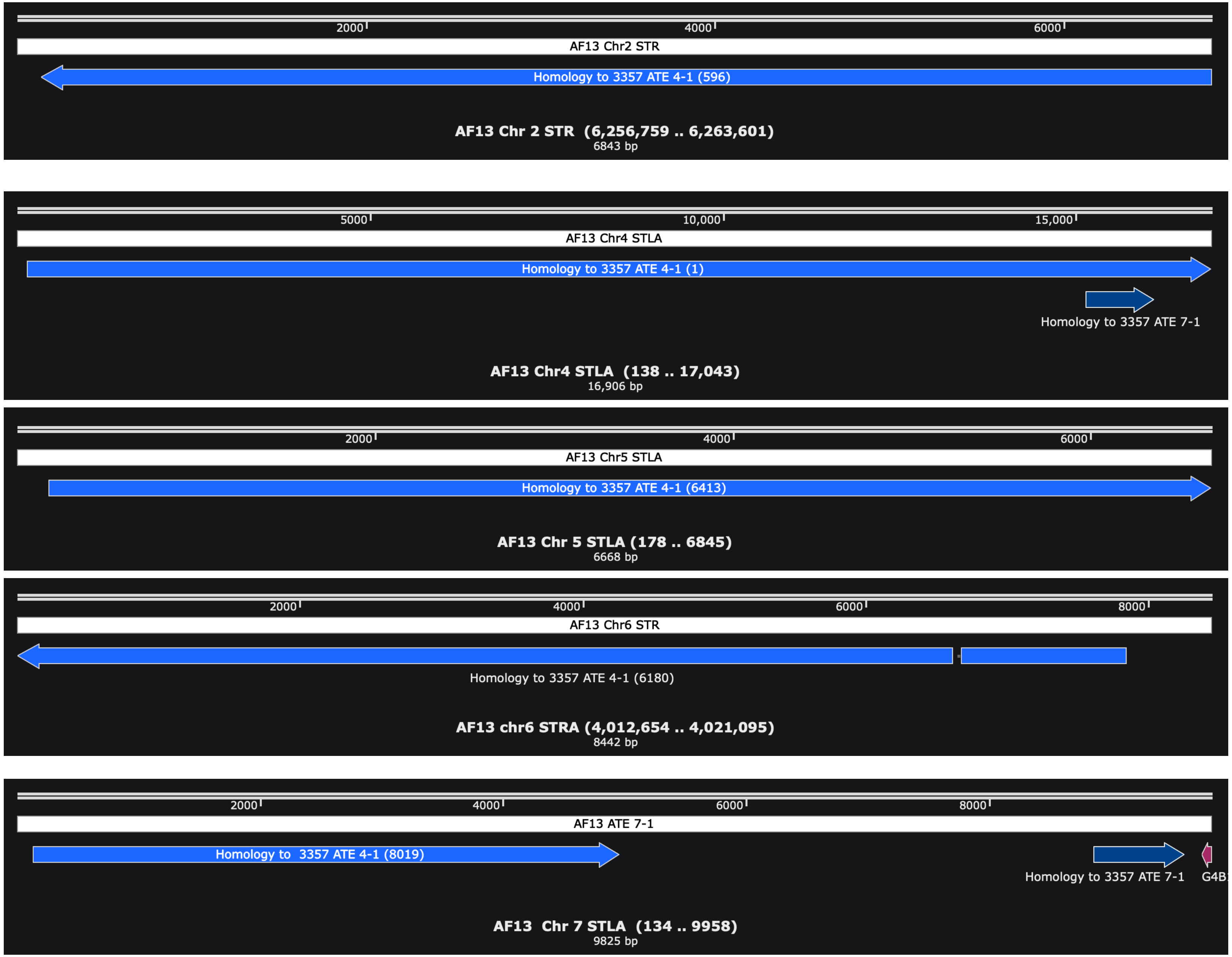

Supplementary Figure 3I: Class I Repeat Organization

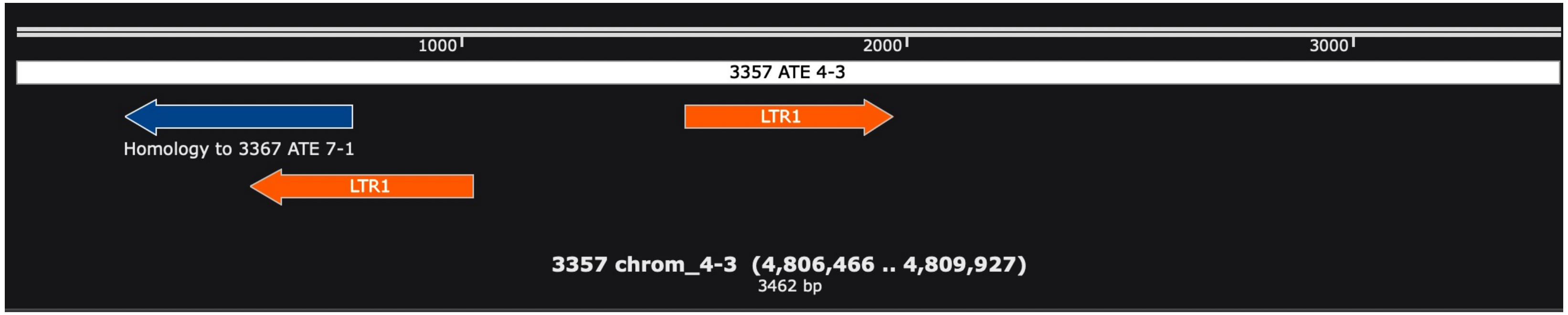

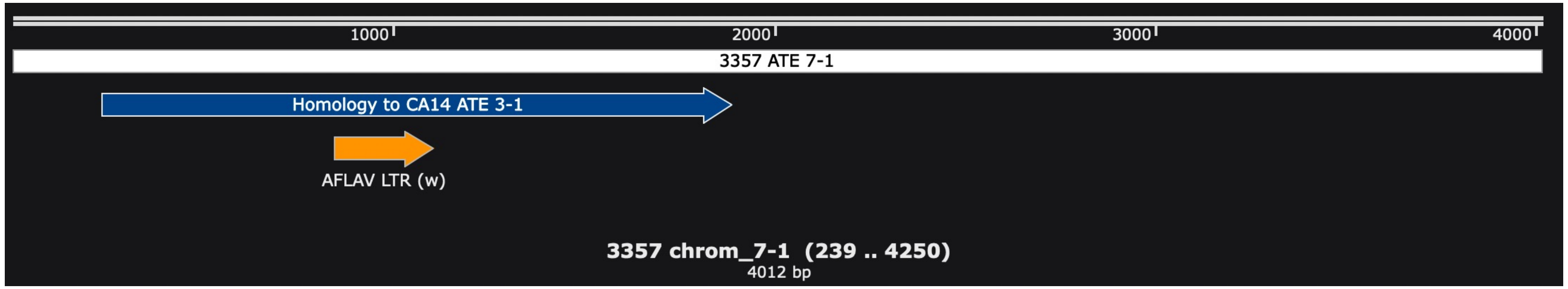

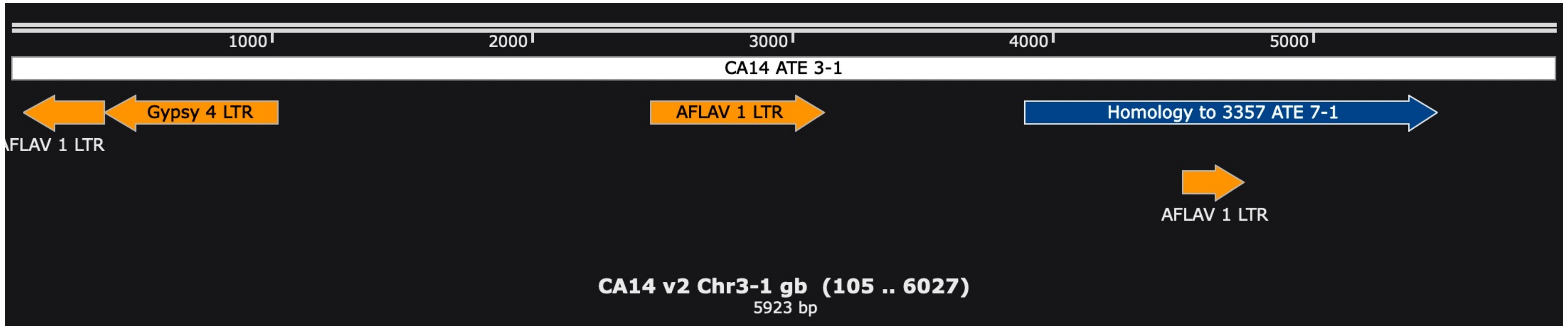

Supplementary Figure 3J: Class J Repeat Organization

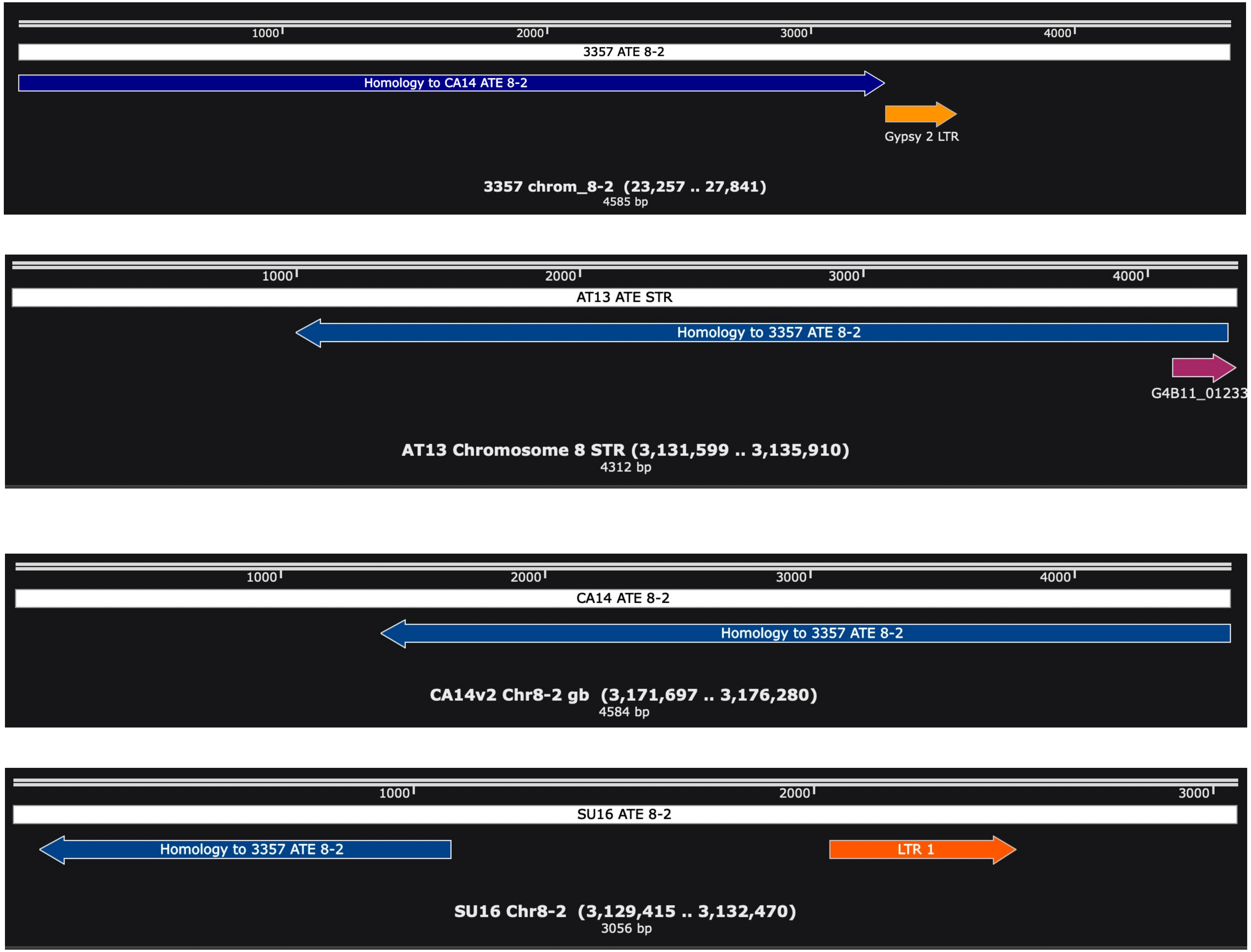

Supplementary Figure 3K: Class K Repeat Organization

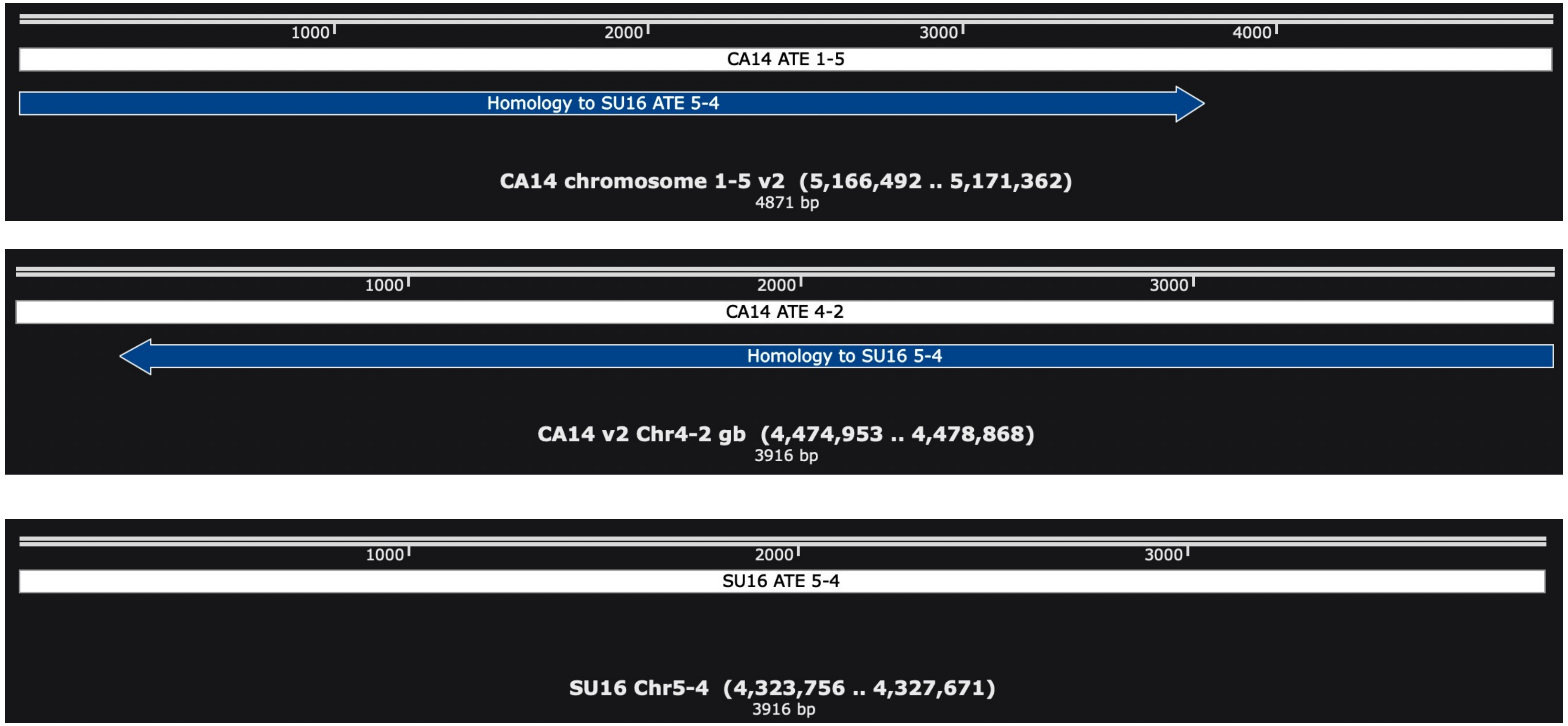

Supplementary Figure 3L: Class L Repeat Organization

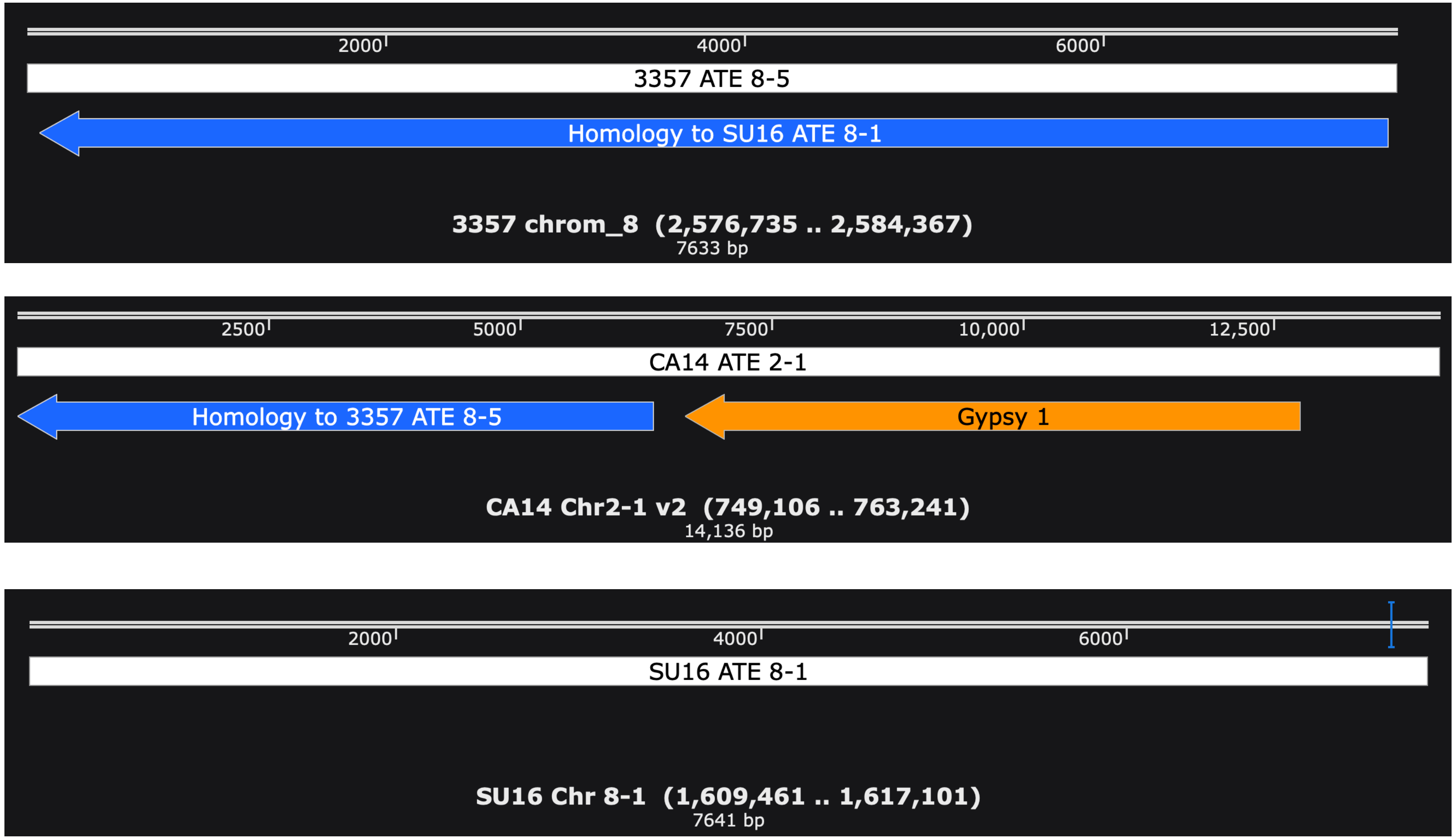

Supplementary Figure 3: Classification of Repeated ATE Homologies into Classes A-L.
