# Supplementary figures and images for: Investigating the origin of subtelomeric and centromeric AT-rich elements in Aspergillus flavus
Source: PLoS One. 2023 Feb 9;18(2):e0279148. doi: 10.1371/journal.pone.0279148 (PMC9910759; doi:10.1371/journal.pone.0279148)

S Fig. 4A

L

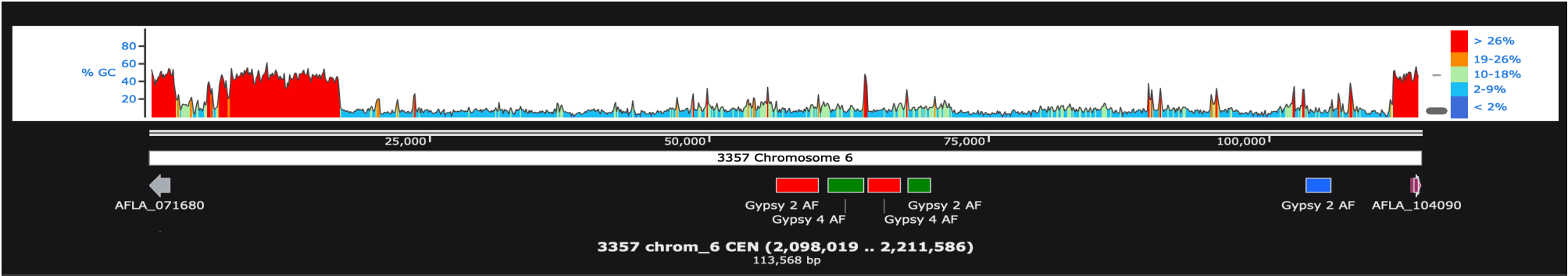

R

R

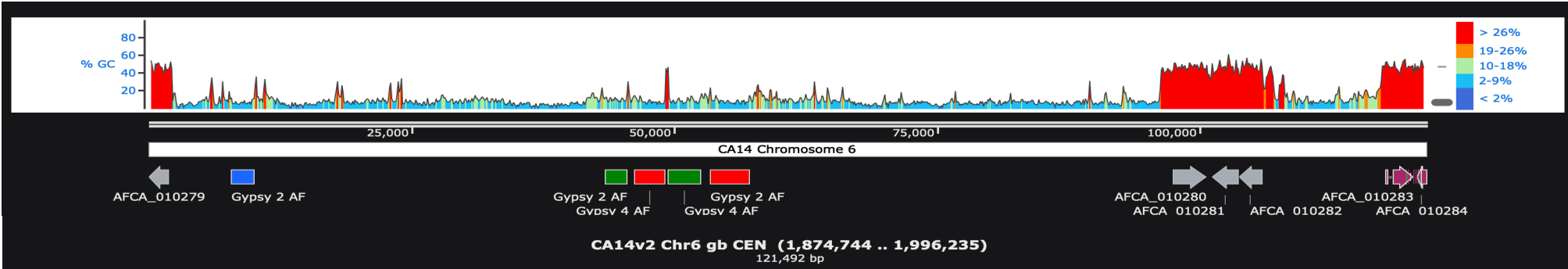

L

L

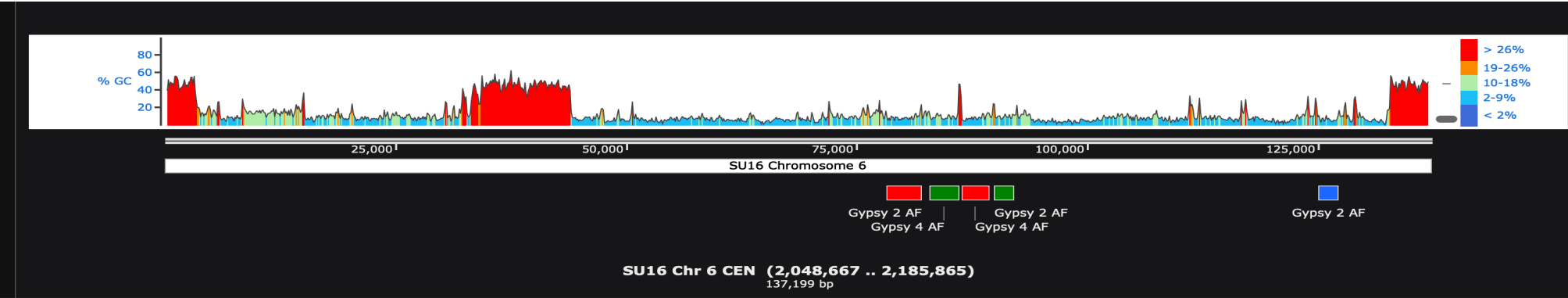

R

Supplement: S4 Fig — The positions of the mutated Gypsy 2 and Gypsy 4 homologs within the centromeric domains of chromosome 6 (A), chromosome 8 (B) and chromosome 1 (C) (between the coordinates listed) were plotted and aligned among the three strains. The red, green, blue, and brown colors refer to regions of high homology as shown in Table 6A and 6B. The white color refers to regions of lower homology [corresponding to the black color in Table 6A and 6B)]. The GC content of these regions is presented above each chromosomal plot to aid in the identification of the centromere. Coding regions flanking the centromere are depicted by the purple arrows. Some chromosomes are inverted relative to left (L) and right (R) arms of NRRL 3357 chromosomes in the NCBI database, as indicated. Specifically, chromosomes 1 and 6 of CA14 are presented in inverted orientations. Chromosome 8 species are presented in inverted orientations in both CA14 and SU-16. (PDF) [file pone.0279148.s004.pdf]
