# Supplementary material for: Investigating the origin of subtelomeric and centromeric AT-rich elements in Aspergillus flavus
Source: PLoS One. 2023 Feb 9;18(2):e0279148. doi: 10.1371/journal.pone.0279148 (PMC9910759; doi:10.1371/journal.pone.0279148)
Supplement: S5 Fig — Sequence analysis of representative H class repeats (a) and chromosome 4 centromeric Gypsy 4 elements (b). (A) BLAST search of NRRL 3357 ATE 1–4 (query) showing a portion of the homology with 3357 ATEs 6–1 (left) and 8–1 (right) (subjects). In this format, mismatches and gaps are represented as red residues. Quantification of the entire repeat in ATE 8–1 displayed 100% transition mutations, while ATE 6–1 displayed 80% transition mutations. All transversion mutations were A/T switches. (B) A portion of chromosome 4 centromeric Gypsy homolog (denoted by the green hue in Table 6 and Fig 3) homology identified in a BLAST search comparing strain NRRL 3357 (query) with strains SU-16 (A) and CA14 (B) (subjects), revealing a high level of sequence conservation. (PDF) [file pone.0279148.s005.pdf]

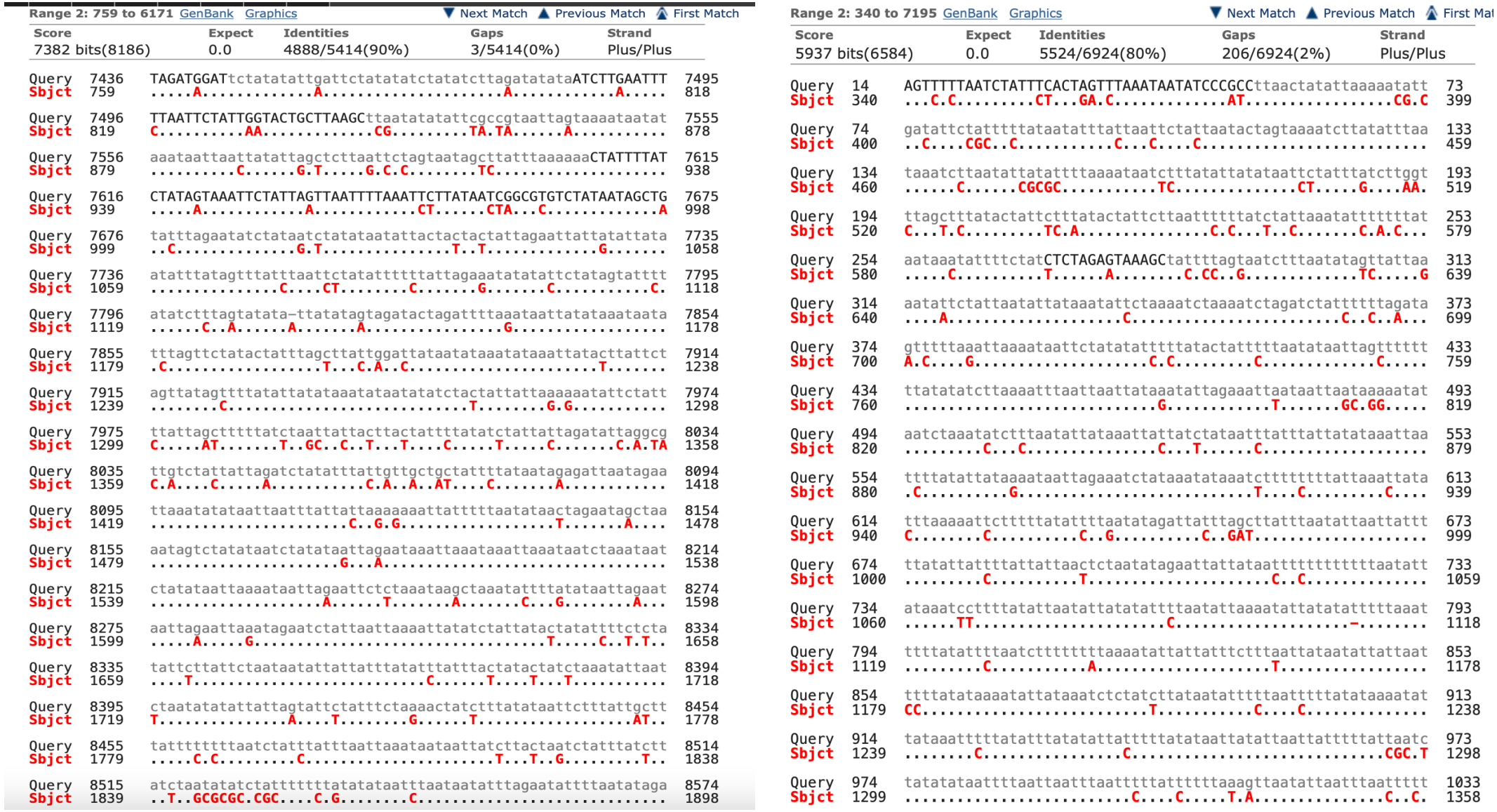

Supplementary Figure 5A. H Class Sequence Homology of NRRL 3357 6L (left) and 8L (right) ATEs with ATE 4-1

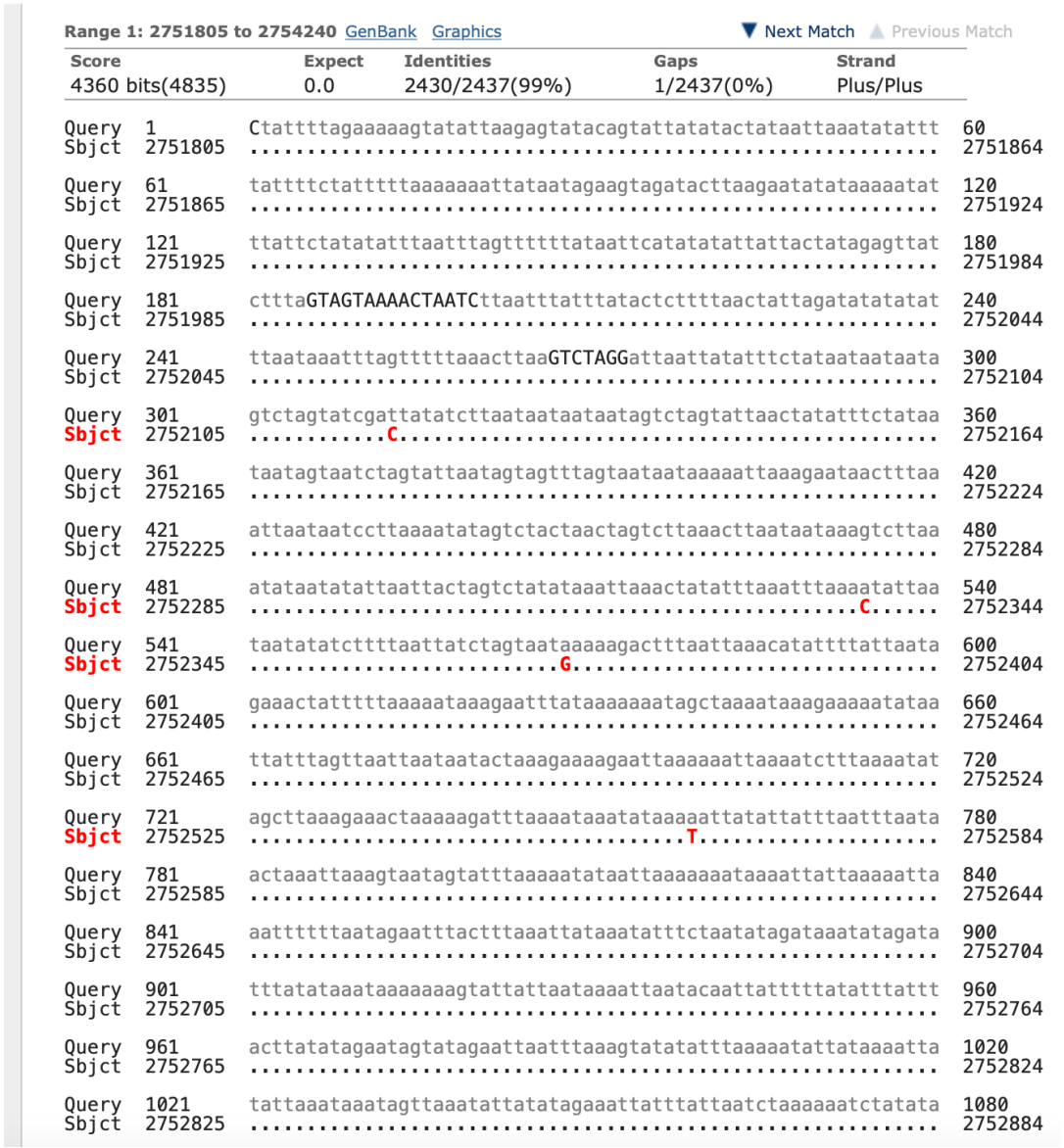

Range 1: 2751805 to 2754240 GenBank Graphics ▼ Next Match ▲ Previous Match

Score	Expect	Identities	Gaps	Strand
4360 bits(4835)	0.0	2430/2437(99%)	1/2437(0%)	Plus/Plus

```
Query  1        Ctattttagaaaaagtatattaagagtatacagtattatatactataattaaatatattt  60
Sbjct  2751805  ............................................................  2751864

Query  61       tattttctatttttaaaaaaattataatagaagtagatacttaagaatatataaaaatat  120
Sbjct  2751865  ............................................................  2751924

Query  121      ttattctatatatttaatttagtttttttataattcatatatattattactatagagttat  180
Sbjct  2751925  ............................................................  2751984

Query  181      ctttaGTAGTAAAACTAATCttaatttatttatactcttttaactattagatatatatat  240
Sbjct  2751985  ............................................................  2752044

Query  241      ttaataaatttagtttttaaacttaaGTCTAGGattaattatatttctataataataata  300
Sbjct  2752045  ............................................................  2752104

Query  301      gtctagtatcgattatatcttaataataataatagtctagtattaactatatttctataa  360
Sbjct  2752105  ...........C................................................  2752164

Query  361      taatagtaatctagtattaatagtagtttagtaataataaaaattaaagaataactttaa  420
Sbjct  2752165  ............................................................  2752224

Query  421      attaataatccttaaaatatagtctactaactagtcttaaacttaataataaagtcttaa  480
Sbjct  2752225  ............................................................  2752284

Query  481      atataatatattaattactagtctatataaattaaactatatttaaatttaaaatattaa  540
Sbjct  2752285  ..........................................................C.....  2752344

Query  541      taatatatcttttaattatctagtaataaaaagactttaattaaacatattttattaata  600
Sbjct  2752345  ........................G...................................  2752404

Query  601      gaaactatttttaaaaataaagaatttataaaaaaatagctaaaataaagaaaaatataa  660
Sbjct  2752405  ............................................................  2752464

Query  661      ttatttagttaattaataatactaaagaaaagaattaaaaaattaaaatctttaaaatat  720
Sbjct  2752465  ............................................................  2752524

Query  721      agcttaaagaaactaaaaagatttaaaataaatataaaaattatattatttaatttaata  780
Sbjct  2752525  ..................................T.........................  2752584

Query  781      actaaattaaagtaatagtatttaaaaatataattaaaaaaataaaattattaaaaatta  840
Sbjct  2752585  ............................................................  2752644

Query  841      aatttttttaatagaatttactttaaattataaatatttctaatatagataaatatagata  900
Sbjct  2752645  ............................................................  2752704

Query  901      tttatataaataaaaaaagtattattaataaaattaatacaattatttttatatttattt  960
Sbjct  2752705  ............................................................  2752764

Query  961      acttatatagaatagtatagaattaatttaaagtatatatttaaaaatattataaaatta  1020
Sbjct  2752765  ............................................................  2752824

Query  1021     tattaaataaatagttaaatattatatagaaattatttattaatctaaaaaatctatata  1080
Sbjct  2752825  ............................................................  2752884
```

Range 1: 2760852 to 2763258 GenBank Graphics ▼ Next Match ▲ Previous Match

Score	Expect	Identities	Gaps	Strand
4044 bits(4484)	0.0	2361/2442(97%)	40/2442(1%)	Plus/Plus

```
Query  1        Ctattttagaaaaagtatattaagagtatacagtattatatactataattaaatatattt  60
Sbjct  2760852  ..............................T.............................  2760911

Query  61       tattttctatttttaaaaaaattataatagaagtagatacttaagaatatataaaaatat  120
Sbjct  2760912  ....................T.....................T.......G.........  2760971

Query  121      ttattctatatatttaatttagtttttttataattcatatatattattactatagagttat  180
Sbjct  2760972  .....T............................A.........................  2761031

Query  181      ctttaGTAGTAAAACTAATCttaatttatttatactcttttaactattagatatatatat  240
Sbjct  2761032  ............................................................  2761091

Query  241      ttaataaatttagtttttaaacttaaGTCTAGGattaattatatttctataataataata  300
Sbjct  2761092  ..............................T.............................  2761151

Query  301      gtctagtatcgattatatcttaataataataatagtctagtattaactatatttctataa  360
Sbjct  2761152  --------------------------------.........G...............  2761177

Query  361      taatagtaatctagtattaatagtagtttagtaataat-aaaaattaaagaataacttta  419
Sbjct  2761178  ........................................A...................  2761237

Query  420      aattaataatccttaaaatatagtctactaactagtcttaaacttaataataaagtctta  479
Sbjct  2761238  .G........T......................A.........................  2761297

Query  480      aatataatatattaattactagtctatataaattaaactatatttaaatttaaaatatta  539
Sbjct  2761298  ............................................................  2761357

Query  540      ataatatatcttttaattatctagtaataaaaagactttaattaaacatattttattaat  599
Sbjct  2761358  ..........................................A.........G...     2761417

Query  600      agaaactatttttaaaaataaagaatttataaaaaaatagctaaaataaagaaaaa-tat  658
Sbjct  2761418  ..........................................G..........T...     2761477

Query  659      aattatttagttaattaataatactaaagaaaagaattaaaaaattaaaatctttaaaat  718
Sbjct  2761478  ............................................................  2761537

Query  719      atagcttaaagaaactaaaaagatttaaaataaatataaaaattatattatttaatttaa  778
Sbjct  2761538  ............................AG..............................  2761597

Query  779      taactaaattaaagtaatagtatttaaaaatataattaaaaaaataaaattattaaaaat  838
Sbjct  2761598  ..G.........................................................C.  2761657

Query  839      taaatttttttaatagaatttactttaaattataaatatttctaatatagataaatataga  898
Sbjct  2761658  ........................C...................................T.  2761717

Query  899      tatttatataaataaaaaaagtattattaataaaattaatacaattatttttatatttat  958
Sbjct  2761718  .......G.....................................G..............  2761777

Query  959      ttacttatatagaatagtatagaattaatttaaagtatatatttaaaaatattataaaat  1018
Sbjct  2761778  ..................................C.........................  2761837

Query  1019     tatattaaataaatagttaaatattatatagaaattatttattaatctaaaaaatctata  1078
Sbjct  2761838  ..............................................T.G...........  2761897
```

Supplementary Figure 5B Gypsy 4 Chr 4 Sequence Comparison Between SU16 (left) or CA14 (right) and NRRL 3357 (query).
