# Supplementary material for: Investigating the origin of subtelomeric and centromeric AT-rich elements in Aspergillus flavus
Source: PLoS One. 2023 Feb 9;18(2):e0279148. doi: 10.1371/journal.pone.0279148 (PMC9910759; doi:10.1371/journal.pone.0279148)
Supplement: S1 Table — Positions of indels are presented using the coordinates of the inserted (top) strain in each strain a/strain b configuration. Red: AT-rich indel, Green: Reiterated AT-rich indel, White: non-AT indel, Blue: Reiterated non-AT indels. Other combinations of strains did not result in the identification of additional elements. Data, after conversion to the relative positions in NRRL 3357, are plotted in Fig 1. (PDF) [file pone.0279148.s006.pdf]

| Deletion/ Insertion | Chr1 site | Chr1 size | Chr2 site | Chr3 size | Chr3 site | Chr3 size | Chr4 site | Chr4 size | Chr5 site | Chr5 size | Chr6 site | Chr6 size | Chr7 site | Chr7 size | Chr8 site | Chr8 size |
|---|---|---|---|---|---|---|---|---|---|---|---|---|---|---|---|---|
| **AF13/AF36** | 296050 | 11991 | 329050 | 11313 | 76250 | 7984 | 4193800 | 12273 | 2837100 | 9687 | 3335650 | 47610 | 1119075 | 9076 | 664356 | 7564 |
| | 607150 | 310114 | 751550 | 17721 | | | 4288450 | 28377 | 3189150 | 10934 | 4000900 | 11767 | | | 2243450 | 6700 |
| | 2452800 | 70805 | 5950050 | 10868 | 1071 | | | | | | | | | | | |
| **AF36/AF13** | | | 812000 | 118447 | | | 4385800 | 7418 | 176710 | 17280 | 428875 | 59719 | 1155000 | 6166 | 1586150 | |
| | | | | | | | | | 4020400 | 21031 | | | | | | |
| | | | | | | | | | | | | | | | | |
| **3357/AF36** | 6155077 | 11984 | 329821 | 11313 | 75559 | 8038 | 215148 | 20131 | 1173910 | 9396 | | | | | | |
| | 6193155 | 14839 | 404773 | 6757 | 955794 | 8491 | 3367581 | 16418 | 1341753 | 10333 | | | | | | |
| | 6471788 | 13711 | 593667 | 32945 | 2688700 | 18140 | 3397136 | 52256 | | | 626897 | 66880 | | | | |
| | | | 3669236 | 9921 | | | 4361220 | 12269 | | | 3147306 | 7369 | | | | |
| | | | | | | | | | | | | | | | | |
| **AF36/3357** | 5827242 | 67701 | 823091 | 107369 | | | 4385854 | 11261 | 4011232 | 32858 | 488605 | 61579 | 144054 | 13458 | | |
| | 3766453 | 31258 | 3003576 | 796803 | | | | | | | | | 439485 | 20085 | | |
| | | | 920762 | 141343 | | | | | | | | | 734063 | 14397 | | |
| | | | | | | | | | | | | | | | | |
| **3357/A9** | 6185032 | 23742 | 404776 | 6758 | 955089 | 9791 | 215146 | 20134 | 182079 | 19080 | 1625834 | 8119 | | | | |
| | 6471794 | 13705 | 593765 | 32817 | | | 3367719 | 18114 | 1173817 | 9138 | 3203199 | 48954 | | | | |
| | | | 3668934 | 9921 | | | 3396805 | 47732 | 2650154 | 9150 | | | | | | |
| | | | 5958294 | 31829 | | | | | | | | | | | | |
| | | | | | | | | | | | | | | | | |
| **A9/3357** | 6733780 | 8095 | 747508 | 17693 | | | 4286632 | 30815 | 2811851 | 15417 | 3294251 | 39534 | | | | |
| | 2935888 | 7341 | 819147 | 20449 | | | 4473738 | 8762 | 3160336 | 16664 | 793000 | 51801 | | | | |
| | 2456426 | 77579 | | | | | | | 3850669 | 11833 | | | | | | |
| | | | | | | | | | | | | | | | | |
| **K49/AF36** | | | | | | | | | | | | | | | | |
| | | | | | | | | | | | | | 47607 | 47462 | | |
| **AF36/K49** | | | | | | | | | | | | | | | | |
| | | | | | | | | | | | | | | | | |
| **Tox4/3357** | | | | | | | | | | | | | 110445 | 10985 | | |
| | | | | | | | | | | | | | | | | |
| **3357/Tox4** | | | | | | | | | | | 3343821 | 38405 | 663854 | 26195 | | |

**Supplementary Table 1.** Positions and lengths of indels.
