# Supplementary material for: Investigating the origin of subtelomeric and centromeric AT-rich elements in Aspergillus flavus
Source: PLoS One. 2023 Feb 9;18(2):e0279148. doi: 10.1371/journal.pone.0279148 (PMC9910759; doi:10.1371/journal.pone.0279148)
Supplement: S2 Table — (PDF) [file pone.0279148.s007.pdf]

CHROMOSOME	# UNIQUE ELEMENTS	MINIMUM INDEL SIZE (bp)	MAXIMUM INDEL SIZE (bp)
**1**	10	7341	323521
**2**	10	6757	796803
**3**	3	8038	18140
**4**	7	7174	52256
**5**	8	9138	32528
**6**	7	7369	32528
**7**	6	6166	6688
**8**	3	6700	9289
**TOTAL**	54	6166	796803

Supplementary Table 2. Summary analysis of 14 strains indicating the number of unique indels and the range of approximate element sizes observed
