# Supplementary material for: Investigating the origin of subtelomeric and centromeric AT-rich elements in Aspergillus flavus
Source: PLoS One. 2023 Feb 9;18(2):e0279148. doi: 10.1371/journal.pone.0279148 (PMC9910759; doi:10.1371/journal.pone.0279148)
Supplement: S3 Table — % AT content, approximate insertion position relative to NRRL 3357, and total fragment length, with the % of the insertion containing the AT-rich sequence in parentheses. CHR, chromosome number. When multiple AT-rich regions are presented within the identified insert, they are separated by semi-colons. (PDF) [file pone.0279148.s008.pdf]

CHR	INSERTED STRAIN	≥ 80% AT (bp)	≥ 70% AT (bp)	≥ 65% AT (bp)	NRRL 3357 POSITION (bp from left arm)	TOTAL FRAG LENGTH (bp) (%AT Length/Total Length)
**1**	AF13	4500; 1700	2500; 3500		4233530	71964 (17.0)
**1**	AF36	2800			2684000	30064 (9.3)
**1**	AF36	7500; 1500			330400	71964 (11.8)
**1**	NRRL 3357		12400		6193155	14227 (87.2)
**1**	A9	4000			41000	21293 (18.8)
**1**	A9			7000	3823000	9019 (77.6)
**2**	AF13	31125			330000	31125 (100)
**2**	AF36		6200		2831000	7802 (79.5)
**2**	NRRL 3357	6300			404773	6787 (92.8)
**2**	NRRL 3357	9500			3669236	10289 (92.3)
**3**	AF13		7321		76250	7321 (100)
**3**	NRRL 3357		3500		2688700	10781 (32.5)
**5**	AF13	5400; 500			2899000	17994 (38.3)
**5**	AF13	3300	3500		3229000	17725 (38.4)
**6**	A9	21000	2000; 11000		3178500	52074 (65.3)
**8**	AF13	7309			126800	7309 (100)

Supplementary Table 3. Tabulation of AT indel insertions in various chromosomes.
