# Supplementary material for: Investigating the origin of subtelomeric and centromeric AT-rich elements in Aspergillus flavus
Source: PLoS One. 2023 Feb 9;18(2):e0279148. doi: 10.1371/journal.pone.0279148 (PMC9910759; doi:10.1371/journal.pone.0279148)
Supplement: S4 Table — AT-indels on each chromosome in a comparison between CA14 and NRRL 3357 genomes. (PDF) [file pone.0279148.s009.pdf]

CHROMOSOME	TOTAL INDELS (#CA14/#NRRL 3357)	AT ELEMENTS (#CA14/#NRRL 3357)
**1**	7	4
**2**	5	4
**3**	1	1
**4**	7	2
**5**	2	0
**6**	2	0
**7**	7	1
**8**	8	3
**TOTAL**	39 (20/19)	15 (10/5)

Supplementary Table 4. Summary of The Total Number of Indels vs. AT-Indels in a Comparison Between CA14 and NRRL 3357 Genomes.
