# Supplementary material for: Investigating the origin of subtelomeric and centromeric AT-rich elements in Aspergillus flavus
Source: PLoS One. 2023 Feb 9;18(2):e0279148. doi: 10.1371/journal.pone.0279148 (PMC9910759; doi:10.1371/journal.pone.0279148)
Supplement: S5 Table — Data is derived from the indel analysis between the two strains, together with the chromosomal location, the approximate length of AT-rich regions, the site of the insertion, and the total length/AT element length % (parentheses) observed in the insertion. Consecutive numbers (separated by a semi-colon) indicate noncontiguous ATEs within the insertion. (PDF) [file pone.0279148.s010.pdf]

AT Indel Number	Location of Chromosome Homology	Strain Containing Insertion	≥ 80% AT	≥ 70% AT	Start Position of AT Insertion (BP)	TOTAL FRAG LENGTH (% of Insertion Containing ATE)
**1-1**	1	NRRL 3357		14000	6193226	14445 (97)
**1-14**	1	NRRL 3357	6500		30815	6763 (96)
**1-4**	1	CA14	7800	1000	1611673	9753 (90)
**1-12**	1	CA14		7900	6463499	8143 (97)
**2-1**	2	NRRL 3357	9805		3669272	9805 (100)
**2-4**	2	NRRL 3357	6500; 1500		404891	21630 (37)
**2-5**	2	NRRL 3357	9400		329839	11138 (84)
**2-3**	2	CA14	6200; 6500		749200	14148 (90)
**3-2**	3	NRRL 3357	6700; 1000; 400		75805	8282 (98)
**4-9**	4	NRRL 3357	9673		7531	9673 (100)
**4-1**	4	CA14	2500	4000; 800; 600	4705672	14113 (56)
**7-6**	7	NRRL 3357	5500		687426	67285 (8)
**8-7**	8	NRRL 3357	7200		2576721	7372 (98)
**8-9**	8	NRRL 3357	7697		2913492	7697 (100)
**8-10**	8	CA14	2200		177065	9151 (24)

Supplementary Table 5. Tabulation of Insertions into CA14 and NRRL 3357 Genomes in Comparisons Between CA14 and NRRL 3357 and the %AT Observed in the Indel
