# Supplementary material for: Investigating the origin of subtelomeric and centromeric AT-rich elements in Aspergillus flavus
Source: PLoS One. 2023 Feb 9;18(2):e0279148. doi: 10.1371/journal.pone.0279148 (PMC9910759; doi:10.1371/journal.pone.0279148)
Supplement: S6 Table — (left) The values of total mismatches/transition mutations (mm/Ts) between the AT-rich homologs and the Gypsy consensus sequence as determined by the Censor program are listed together with their standard deviation (SD) and sample size (n). (right) The fraction of the total transition mutations accounted for by G>A and C>T mutations within the consensus sequence was determined as described in Materials and Methods. Both full length and deleted forms of candidates were analyzed; nm, not measured. (PDF) [file pone.0279148.s011.pdf]

TE Class	Mm/Ts<br>Average ± SD (n)	% Ts G>A, C>T<br>Average ± SD (n)
**Gypsy 4 LTR**	1.0358 ± 0.050 (27)	0.97± 0.05 (8)
**Gypsy 1 LTR**	1.0319± 0.038 (22)	0.99 ± 0.01 (15)
**Gypsy 2 LTR**	nm	0.8 (1)
**Gypsy 4 AO**	1.0103 ± 0.008 (8)	0.99 ± 0.004 (2)
**Gypsy 1 AO**	1.0047 ± 0.005 (8)	0.99 ± 0.001 (2)
**Gypsy 2 AO**	1.0072 ± 0.009 (2)	0. 99± 0.007 (2)

Supplementary Table 6. Mutations in Gypsy homologs are characteristic of RIP.
