# Supplementary material for: Investigating the origin of subtelomeric and centromeric AT-rich elements in Aspergillus flavus
Source: PLoS One. 2023 Feb 9;18(2):e0279148. doi: 10.1371/journal.pone.0279148 (PMC9910759; doi:10.1371/journal.pone.0279148)
Supplement: S1 Fig — Strains were compared by the Blast Tree Widget using the Fast Minimum Evolution algorithm, filtered at 90% homology. The tree is rooted in CA14 non-AT insertion 1–11. The scale for 0.009% homology difference is shown on the bottom right. (PDF) [file pone.0279148.s001.pdf]

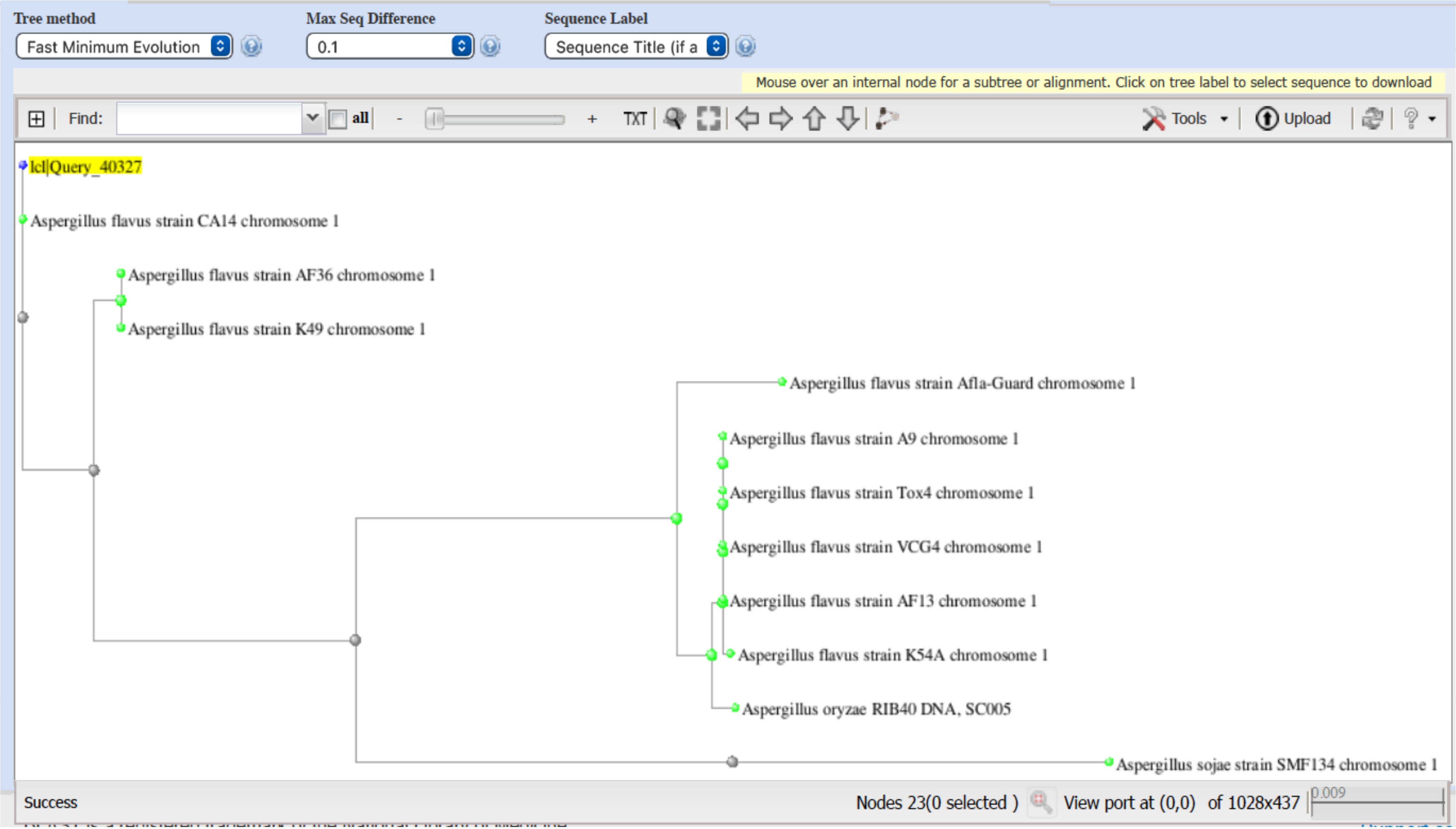

Supplementary Figure 1: CA14 Chr1-11 Non-AT Indel Phylogenetic Comparisons with Other Strain Displaying Strains at the 90% Homology Level. The scale for 0.009% homology difference is shown on the bottom right. Tree is rooted in CA14.
