# Supplementary material for: Investigating the origin of subtelomeric and centromeric AT-rich elements in Aspergillus flavus
Source: PLoS One. 2023 Feb 9;18(2):e0279148. doi: 10.1371/journal.pone.0279148 (PMC9910759; doi:10.1371/journal.pone.0279148)
Supplement: Attachment — Submitted filename: Responsa Letter .docx [file pone.0279148.s012.docx]

Response to Reviewers:

I would like to thank the editor and reviewers for their critique of the manuscript. I have attempted to address each issue as enumerated below. **The page and line designations in the revised text refer to the marked up manuscript and are indicated in the bold font**.

*General Comment on Figure Resolution and Textual Clarity*:

Although the figures passed the PACE pre-flight analysis, I have increased the resolution of Figures 3-5 from 300 to 400 dpi. I have also increased the text size in Figures 3, 4, and 5 so that they are more legible when reduced in size.

Beyond the changes made to the text based on the critiques, the text has also been slightly modified for clarity in reading as indicated in the markup

Reviewer 1:

General Comments

1. I have added a new Table 3 (**Line 461**) that describes the class designations for Classes A-L (see note below) as requested.

To correlate the Abstract more carefully with Results, I have modified the text (**Lines 25-40**) of the Abstract as requested so that the first two categories of ATE classes in non-centromeric regions are contiguous and specifically list classes A through J. [Classes K and L are not discussed in the Abstract. I have also eliminated the description of the term “classes” in the Abstract and Introduction as they related to types of ATEs with “categories” to eliminate confusion.

I have also discussed the homolog classes in the section “Identification of multiple classes of homologous AT elements” and describe which classes are found in each strain as they relate to Tables 2A, 2B and 2C (**Lines 389-398**).

[Note: The addition of Class L results from the two additional NRRL 3357 (ATE 8-5 and 8-6 in Table 2A) erroneously omitted from the original manuscript. The number of ATEs in NRRL 3357 has been adjusted accordingly in the text [**Lines 341, 344, 345, 390**]. Class L, found in three ATEs, is qualitatively like Class K (no identifiable TE element, not in subtelomeric regions, present in many A. flavus and A. oryzae strains) is discussed at the end of the Results sections (**Lines 648-653**). Class L has also been added to Supplementary Figure 3.

Specific Comments:

1. Line 66: The Selker et al reference has been added to the RIP references. I have clarified that RIP occurs in pre-meiotic stages, rather than in a specific stage of meiosis. **Line 74. Reference 26.**
2. Line 80: Silencer information regulators (SIR) proteins are silencing- and heterochromatic-related proteins first identified in budding yeast (as referenced now) and subsequently in other organisms (**Line 88 reference 34**).
3. Line 92: Plasmodia has been changed to Trypanosoma (**Line 100**).
4. Line 107. I now state that “each strain has a different ATE size…”(**Line 115**).
5. Line110: The phrase “non-centromeric” has been removed (**Line 118-119**)
6. Line 124: The four sequenced genomes (NRRL 2999), VCG1, VCG4, and A1) with their accession numbers and the published reference are now presented (**Lines 133-135 reference 45**).
7. Line 202: I explain that RIP results from “a cytosine residue … mutating to a thymidine (and the corresponding guanine to adenine mutation on the complementary strand),”…(**Lines 214-216**).
8. Line 253: The Reviewer is correct that major (suggesting a majority) should be eliminated. I replaced it with “significant” (**Line 265**).
9. Line 302: “Strains” were replaced with “genomes”: “First, non-AT insertions from the genomes of related strains were most often located (in 20/24 cases) at allelic positions in non-AT insertions” (**Line 323; also 305-320**).

Line 304, I have deleted “in different strains” (**Line 326**).

10. Line 321: The subtelomeric regions are indicated by the yellow shaded entries in the box (not by red font) as is now stated (**Line 343**).
11. Line 363: I have corrected the indicated sentence to state: “Reanalysis of AT-element size NRRL 3357 led to a revised size ranges from 1.9 kb-21.2 kb (**Lines 390-391**).
12. Line 369: The conclusion omits that differing ATE element size is a major difference between the genomes of the strains. Rather, I state “These data revealed that ATE length and chromosomal positions varied significantly among the different genomes.” (**Lines 396-398**).
13. Line 431: I did not intend to convey the impression that RIP is always context dependent. I now state: “RIP activity can depend in some fungi on the local context of the mutated cytosine residues and ultimately gives rise to AT-rich sequences” (**Line 488**)
14. Line 427: Both RIPcal and RIPper are great tools for genome analysis over longer domains they are limited in context of these studies. I state in the revised text:” …RIPper and RIPCAL [51, 57], two algorithms for measuring likely RIP frequencies over genomic distances, do not provide interpretable information for the relatively short and highly mutated sequences under analysis here” (**Lines 482-485**).
15. Line 566: “Flavis” has been corrected to “Flavi”, the name of the clade containing several species related to flavus (**Line 630**).
16. Line 618: Neurospora has now been italicized (**Line 684**).

Table 5A, B (now Table 6):

1. We now state explicitly that the second column refers to the strain containing the Gypsy element used for comparison in the analysis (**Lines 563-564, 574-575**) We have corrected line 9 of Figure 6A to the appropriate NRRL 3357 standard of 100% . In addition, the term “NRRL” has been added as requested.
2. EP is noted only in the strain used for comparison. I have therefore deleted the phrase “in all three strains” (**Lines 563, 573**).

Supplementary Table 3

1. I had used the slash as an indication of two non-contiguous fragments in the AT-rich region as better stated in the Legend to Supplementary Table 5. However, the slash looks like a fraction and have therefore eliminated them from both Tables with an explanation that the semi-colon refers to non-contiguous regions (**Lines 1050-1051, 1060**).

**Reviewer # 2:**

General comments:

The original indel analysis provided the logic for the search for ATEs and the initial data for the dispersion of the ATEs. I have tried to specify the relationship more clearly between the indel and the ATE searches by noting each of the ATEs that were first found by the indel analysis in Tables 2A and 2B as marked by the hashtag (indicated in the Table legends) (**Lines 361, 369-370**). In addition, I have added a line in the text stating “that most of the insertions identified in the indel analysis between CA14 and NRRL 3357 (indicated by the hashtag in Tables 2A and 2B) were also found in these more detailed analyses (**Lines 350-352**). I have also supplied the coordinates for the insertions found from the indel analysis in Supplementary Table 3 so that they can be compared with the coordinates for the ATEs in Table 2.

Specific Comments:

1. In the revised text I have provided information regarding the total size of the genome, the range of sizes of chromosomes 1-8 and the estimated number of genes (13,500) in the first paragraph (**Lines 43-47**): “*Aspergillus flavus* is an opportunistic pathogen in plants and humans. Its 37.5 Mb genome is separated into 8 chromosomes ranging in size from 6.5 Mb of chromosome 1 to 3.25 Mb of chromosome 8. The organization of *Aspergillus* genomes is unusual. Like many fungi, the genome is relatively compact containing approximately 13,500 genes in its genome [1] (on average, 1 gene per 3 kb of DNA).” For simplicity, the subtelomeric regions are defined operationally as the terminal 100 kb at each of the chromosomes.
2. I agree that Table 1 is confusing as presented and have reconfigured both the Table and the Table Legend. The first column represents individual inserts from NRRL 3357, and the other columns represent the chromosome that contains a homolog in the genomes of both NRRL 3357 and/or other strains. I have now separated the previous yellow regions into two subsets. The Xs indicated by a yellow shading are insertions presented at the allelic site in the same chromosome in both NRRL 3357 and other strains. Those indicated by the blue shade are insertions at non-allelic sites at one or more positions within other chromosomes. Those X that are unshaded are insertions in strains other than NRRL 3357. The textual changes are described in **Lines 305-3200.**
3. The text describing Table 1 has been changed to state: “First, non-AT insertions from the genomes of related strains were most often located (in 20/24 cases)” (**Line 323**).
4. Table 2A, 2B, and 2C. I have eliminated the Table description of TEs within each ATE and pooled that data in an extended Table 4A (**Line 467**) (changed from Table 3A by the addition of a new Table 3). ”TAD” has been changed to “TAD1”
5. A clarification of the classes is provided by textually describing the classes found in each strain in Tables 2A, 2B, and 2C. Note the addition of a new Class L as indicated by the note to Reviewer 1. A summary of each class is also provided by a new Table, Table 3 (**Line 461**) that should enhance textual understanding.
6. In the Discussion, the manner in which ATEs may protect against transposition is now stated directly: “One of the functions of ATE formation is the protection against the damaging effects of transposition through inactivation of these elements by RIP activity. However, my analysis suggests that these regions have evolved to perform new functions, at least at telomeres and centromeres.” (**Line 694-695**).
